# Supplementary material for: Antibiotic prescribing for lower UTI in elderly patients in primary care and risk of bloodstream infection: A cohort study using electronic health records in England
Source: PLoS Med. 2020 Sep 21;17(9):e1003336. doi: 10.1371/journal.pmed.1003336 (PMC7505443; doi:10.1371/journal.pmed.1003336)
Supplement: S1 Table — ICD-10, International Classification of Diseases 10th revision. (DOCX) [file pmed.1003336.s002.docx]

**S1 Table –** Read codes and ICD-10 codes used to define study population, exposures, outcomes and covariates. ICD-10, International Classification of Diseases 10th revision.

All code lists are based on Gharbi et al. (2019) (see main reference list). Were changes were made to the code lists, these are indicated at the corresponding table. All tables are also available as Excel workbooks at <https://github.com/prockenschaub/CPRD_UTI_sepsis_elderly>.

| **Lower UTI** | **Primary care** |
| --- | --- |
| Read code | Description |
| 1AG..00 | Recurrent urinary tract infections |
| 1J4..00 | Suspected UTI |
| K15..00 | Cystitis |
| K150.00 | Acute cystitis |
| K15z.00 | Cystitis NOS |
| K190.00 | Urinary tract infection, site not specified |
| K190.11 | Recurrent urinary tract infection |
| K190100 | Pyuria, site not specified |
| K190200 | Post operative urinary tract infection |
| K190300 | Recurrent urinary tract infection |
| K190311 | Recurrent UTI |
| K190400 | Chronic urinary tract infection |
| K190500 | Urinary tract infection |
| K190z00 | Urinary tract infection, site not specified NOS |
| SP07700 | Infect+inflam react due pros dev, implt+graft in urinary syst |
| SP07Q00 | Catheter associated urinary tract infection |
| SP07Q11 | CAUTI - catheter associated urinary tract infection |
| K15y.00 | Other specified cystitis |
| K152z00 | Other chronic cystitis NOS |
| K152.00 | Other chronic cystitis |
| K152y00 | Chronic cystitis unspecified |
| K155.00 | Recurrent cystitis |
| K15yz00 | Other cystitis NOS |
| 1AZ6000 | Mild lower urinary tract symptoms |
| 7N51.00 | [SO]Lower urinary tract |
| 1AZ6100 | Moderate lower urinary tract symptoms |
| 1AZ6.00 | Lower urinary tract symptoms |
| Kyu5100 | [X]Other cystitis |
| 14D4.00 | H/O: recurrent cystitis |
|  |  |
| **Upper UTI** | **Primary care** |
| Read code | Description |
| K100.00 | Chronic pyelonephritis |
| K100000 | Chronic pyelonephritis without medullary necrosis |
| K100100 | Chronic pyelonephritis with medullary necrosis |
| K100400 | Nonobstructive reflux-associated chronic pyelonephritis |
| K100500 | Chronic obstructive pyelonephritis |
| K100600 | Calculous pyelonephritis |
| K100z00 | Chronic pyelonephritis NOS |
| K101.00 | Acute pyelonephritis |
| K101000 | Acute pyelonephritis without medullary necrosis |
| K101z00 | Acute pyelonephritis NOS |
| K104.00 | Xanthogranulomatous pyelonephritis |
| K10y.00 | Pyelonephritis and pyonephrosis unspecified |
| K10y000 | Pyelonephritis unspecified |
| K10y300 | Pyelonephritis in diseases EC |
| K10yz00 | Unspecified pyelonephritis NOS |
| K100200 | Chronic pyelitis |
| K10y400 | Pyelitis in diseases EC |
| K101200 | Acute pyelitis |
| K10y100 | Pyelitis unspecified |
| K102000 | Renal abscess |
| K102.00 | Renal and perinephric abscess |
| K102100 | Perinephric abscess |
| K102z00 | Renal and perinephric abscess NOS |
| K10..00 | Infections of kidney |
| K10z.00 | Infection of kidney NOS |
| K10..11 | Renal infections |
| K10..00 | Infections of kidney |
| K10z.00 | Infection of kidney NOS |
| K21..11 | Prostatitis and other inflammatory diseases of prostate |
| K210.00 | Acute prostatitis |
| K211.00 | Chronic prostatitis |
| K214.00 | Prostatitis in diseases EC |
| K214z00 | Prostatitis in diseases EC NOS |
| K21z.00 | Prostatitis NOS |
| K213.00 | Prostatocystitis |
| K212.00 | Abscess of prostate |
| K10y200 | Pyonephrosis unspecified |
| K105.00 | Chronic infective interstitial nephritis |
| A160200 | Tuberculous pyelonephritis |
| A160100 | Tuberculous pyelitis |
|  |  |
| **Recurrent UTI** | **Primary care** |
| Read code | Description |
| 1AG..00 | Recurrent urinary tract infections |
| K190.11 | Recurrent urinary tract infection |
| K190300 | Recurrent urinary tract infection |
| K190311 | Recurrent UTI |
| K190400 | Chronic urinary tract infection |
| K152z00 | Other chronic cystitis NOS |
| K152.00 | Other chronic cystitis |
| K152y00 | Chronic cystitis unspecified |
| K155.00 | Recurrent cystitis |
| 14D4.00 | H/O: recurrent cystitis |
| K100.00 | Chronic pyelonephritis |
| K100000 | Chronic pyelonephritis without medullary necrosis |
| K100100 | Chronic pyelonephritis with medullary necrosis |
| K100400 | Nonobstructive reflux-associated chronic pyelonephritis |
| K100500 | Chronic obstructive pyelonephritis |
| K100600 | Calculous pyelonephritis |
| K100z00 | Chronic pyelonephritis NOS |
| K100200 | Chronic pyelitis |
| K211.00 | Chronic prostatitis |
| K105.00 | Chronic infective interstitial nephritis |
|  |  |
| **BSI** | **Primary care** |
| readcode | description |
| A38z.11 | Sepsis |
| A38..00 | Septicaemia |
| A3C..00 | Sepsis |
| K190600 | Urosepsis |
| A38z.00 | Septicaemia NOS |
| A381.00 | Staphylococcal septicaemia |
| R106.00 | [D]Unspecified bacteraemia |
| A380.00 | Streptococcal septicaemia |
| A382.00 | Pneumococcal septicaemia |
| A384200 | Escherichia coli septicaemia |
| A384211 | E.coli septicaemia |
| A384.00 | Septicaemia due to other gram negative organisms |
| A3Cz.00 | Sepsis NOS |
| A3Cy.00 | Other specified sepsis |
| A381000 | Septicaemia due to Staphylococcus aureus |
| A38y.00 | Other specified septicaemias |
| A380100 | Septicaemia due to streptococcus, group B |
| A384300 | Pseudomonas septicaemia |
| A384000 | Gram negative septicaemia NOS |
| A380300 | Septicaemia due to streptococcus pneumoniae |
| A384100 | Haemophilus influenzae septicaemia |
| A380400 | Septicaemia due to enterococcus |
| A380000 | Septicaemia due to streptococcus, group A |
| Ayu3J00 | [X]Septicaemia, unspecified |
| AB2y300 | Candidal septicaemia |
| A270100 | Listeria septicaemia |
| A383.00 | Septicaemia due to anaerobes |
| A3C3.00 | Sepsis due to Gram negative bacteria |
| A381100 | Septicaemia due to coagulase negative staphylococcus |
| A3C0100 | Sepsis due to Streptococcus group B |
| A380500 | Vancomycin resistant enterococcal septicaemia |
| A3C1.00 | Sepsis due to Staphylococcus |
| A384400 | Serratia septicaemia |
| A3C2.11 | Sepsis due to anaerobes |
| AB2y500 | Candidal sepsis |
| A3C1000 | Sepsis due to Staphylococcus aureus |
| A3C0300 | Sepsis due to Streptococcus pneumoniae |
| A3C0000 | Sepsis due to Streptococcus group A |
| A3C0.00 | Sepsis due to Streptococcus |
| A270611 | Listerial sepsis |
| A384z00 | Other gram negative septicaemia NOS |
| A3C0z00 | Streptococcal sepsis, unspecified |
| A3C0y00 | Other streptococcal sepsis |
| Ayu3F00 | [X]Streptococcal septicaemia, unspecified |
| A396.00 | Sepsis due to Actinomyces |
| A3C2.00 | Sepsis due to anaerobic bacteria |
| Ayu3E00 | [X]Other streptococcal septicaemia |
| A271100 | Erysipelothrix septicaemia |
| Ayu3G00 | [X]Septicaemia due to other gram-negative organisms |
| A3C3.11 | Sepsis due to Gram negative organisms |
| AB2y511 | Sepsis due to Candida |
| A3C3y00 | Sepsis due to other Gram negative organisms |
| A270600 | Sepsis due to Listeria monocytogenes |
| Ayu3H00 | [X]Other specified septicaemia |
|  |  |
| **Lower UTI** | **Secondary care** |
| ICD-10 | Description |
| N30.0 | Acute cystitis |
| N30.9 | Cystitis, unspecified |
| N30.8 | Other cystitis |
| N39.0 | Urinary tract infection, site not specified |
|  |  |
| **Upper UTI** | **Secondary care** (excluding urinary calculus) |
| ICD-10 | Description |
| N10 | Acute tubulo-interstitial nephritis |
| N12 | Tubulo-interstitial nephritis, not specified as acute or chronic |
| N13.6 | Pyonephrosis |
| N15.1 | Renal and perinephric abscess |
| N15.8 | Other specified renal tubulo-interstitial diseases |
| N15.9 | Renal tubulo-interstitial disease, unspecified |
| N16.0 | Renal tubulo-interstitial disorders in infectious and parasitic diseases classified elsewhere |
| N28.8 | Other specified disorders of kidney and ureter |
| N34.0 | Urethral abscess |
| N34.1 | Nonspecific urethritis |
| N34.2 | Other urethritis |
| N34.3 | Urethral syndrome, unspecified |
| N41.0 | Acute prostatitis |
| N41.1 | Chronic prostatitis |
| N41.2 | Abscess of prostate |
| N41.3 | Prostatocystitis |
| N41.8 | Other inflammatory diseases of prostate |
| N41.9 | Inflammatory disease of prostate, unspecified |
| N11.0 | Nonobstructive reflux-associated chronic pyelonephritis |
| N11.1 | Chronic obstructive pyelonephritis |
| N11.8 | Other chronic tubulo-interstitial nephritis |
| N11.9 | Chronic tubulo-interstitial nephritis, unspecified |
|  |  |
| **BSI** | **Secondary care** (excluding R78.81, which did not exist in our dataset) |
| ICD-10 | Description |
| A40.0 | Sepsis due to streptococcus, group A |
| A40.1 | Sepsis due to streptococcus, group B |
| A40.2 | Sepsis due to streptococcus, group D |
| A40.3 | Sepsis due to Streptococcus pneumoniae |
| A40.8 | Other streptococcal sepsis |
| A40.9 | Streptococcal sepsis, unspecified |
| A41.0 | Sepsis due to Staphylococcus aureus |
| A41.1 | Sepsis due to other specified staphylococcus |
| A41.2 | Sepsis due to unspecified staphylococcus |
| A41.3 | Sepsis due to Haemophilus influenzae |
| A41.4 | Sepsis due to anaerobes |
| A41.5 | Sepsis due to other Gram-negative organisms |
| A41.8 | Other specified sepsis |
| A41.9 | Sepsis, unspecified |
| R57.2 | Septic shock |
| R65.0 | Severe sepsis without septic shock |
| R65.1 | Severe sepsis with septic shock |

**Note**: Due to the large number of ICD-10 codes for other infections, the list of codes used to identify “other infectious cause” in Table 3 are not included in this document. Please refer to <https://github.com/prockenschaub/CPRD_UTI_sepsis_elderly> for a list.
