# Supplementary material for: Antibiotic prescribing for lower UTI in elderly patients in primary care and risk of bloodstream infection: A cohort study using electronic health records in England
Source: PLoS Med. 2020 Sep 21;17(9):e1003336. doi: 10.1371/journal.pmed.1003336 (PMC7505443; doi:10.1371/journal.pmed.1003336)
Supplement: S2 Table — BSI, bloodstream infection; UTI, urinary tract infection. (DOCX) [file pmed.1003336.s003.docx]

**S2 Table -** Generalized estimating equation models of the association between immediate antibiotic prescribing for UTI and BSI in women. BSI, bloodstream infection; UTI, urinary tract infection.

|  |  |  |  |  |  |
| --- | --- | --- | --- | --- | --- |
|  | **Univariable analysis** | |  | **Multivariable analysis*** | |
| **Patient characteristics** | OR (95% CI) | p-value |  | aOR (95% CI) | p-value |
|  |  |  |  |  |  |
|  |  |  |  |  |  |
| **No antibiotic** | 1.72 (1.41-2.11) | <0.001 |  | 1.27 (1.03-1.57) | 0.024 |
|  |  |  |  |  |  |
| **Age** (continuous; per 5 years) | 1.38 (1.32-1.43) | <0.001 |  | 1.24 (1.19-1.31) | <0.001 |
| **IMD**  Q1 (least deprived) | 1 |  |  | 1 |  |
| Q2 | 1.42 (1.13-1.79) | 0.003 |  | 1.36 (1.08-1.71) | 0.010 |
| Q3 | 1.56 (1.24-1.96) | <0.001 |  | 1.43 (1.13-1.80) | 0.003 |
| Q4 | 1.50 (1.17-1.92) | 0.001 |  | 1.37 (1.06-1.76) | 0.015 |
| Q5 (most deprived) | 1.82 (1.40-2.35) | <0.001 |  | 1.49 (1.14-1.94) | 0.003 |
| **Region**  South of England | 1 |  |  | 1 |  |
| London | 1.01 (0.77-1.33) | 0.926 |  | 0.95 (0.72-1.26) | 0.715 |
| Midlands and east of England | 1.15 (0.96-1.38) | 0.123 |  | 1.10 (0.92-1.32) | 0.288 |
| North of England and Yorkshire | 1.21 (1.00-1.47) | 0.053 |  | 1.07 (0.88-1.31) | 0.485 |
| **NHS financial year**  2007/08 | 1 |  |  | 1 |  |
| 2008/09 | 1.03 (0.76-1.40) | 0.857 |  | 1.03 (0.75-1.40) | 0.871 |
| 2009/10 | 0.90 (0.66-1.23) | 0.496 |  | 0.88 (0.64-1.21) | 0.430 |
| 2010/11 | 1.12 (0.83-1.51) | 0.468 |  | 1.09 (0.80-1.48) | 0.594 |
| 2011/12 | 1.08 (0.80-1.46) | 0.606 |  | 1.06 (0.77-1.44) | 0.733 |
| 2012/13 | 1.30 (0.98-1.74) | 0.073 |  | 1.28 (0.94-1.73) | 0.113 |
| 2013/14 | 1.36 (1.01-1.82) | 0.042 |  | 1.33 (0.98-1.81) | 0.063 |
| 2014/15 | 1.83 (1.36-2.45) | <0.001 |  | 1.77 (1.30-2.40) | <0.001 |
| **CCI** (continuous)^†^ | 1.94 (1.77-2.13) | <0.001 |  | 1.51 (1.36-1.67) | <0.001 |
| **Smoking status**  Non-smoker | 1 |  |  | 1 |  |
| Ex-smoker | 0.96 (0.81-1.13) | 0.609 |  | 0.94 (0.79-1.12) | 0.495 |
| Smoker | 1.09 (0.82-1.45) | 0.543 |  | 1.34 (1.00-1.80) | 0.046 |
| **Hospital stays**  Discharged from hospital in prior 7 days | 3.18 (2.28-4.43) | <0.001 |  | 1.34 (0.88-2.03) | 0.177 |
| Discharged from hospital in prior 30 days | 2.65 (2.15-3.27) | <0.001 |  | 1.38 (1.03-1.83) | 0.029 |
| Number of days spent in hospital  in prior year^†^ | 1.23 (1.20-1.26) | <0.001 |  | 1.07 (1.02-1.12) | 0.003 |
| Number of admissions in prior year^†^ | 2.44 (2.20-2.70) | <0.001 |  | 1.50 (1.21-1.87) | <0.001 |
| **A&E attendances**  A&E attendance in prior 30 days | 2.51 (1.92-3.29) | <0.001 |  | 1.18 (0.84-1.66) | 0.346 |
| Number of attendances in prior year^†^ | 1.79 (1.63-1.96) | <0.001 |  | 0.92 (0.78-1.09) | 0.340 |
| **Antibiotic in prior 30 days** | 1.48 (1.25-1.75) | <0.001 |  | 1.21 (1.02-1.44) | 0.029 |
| **Index event was home visit** | 4.06 (3.31-4.99) | <0.001 |  | 2.19 (1.75-2.75) | <0.001 |
|  |  |  |  |  |  |

A&E, accident and emergency; aOR, adjusted odds ratio; CCI, Charlson Comorbidity Index; IMD, Index of Multiple Deprivation 2015; NHS, UK National Health Service; OR, crude odds ratio; Q1–Q5, quintiles 1–5; UTI, urinary tract infection; 95% CI, 95% confidence interval.

* adjusted for all other variables included in the table

^†^ Transformed using the square root before input into the model. Effect sizes represent the relative change in odds (OR) *per 1 unit increase in the square root*, that is when the risk factor increases from 0 to 1, from 1 to 4, from 4 to 9, etc. on the original scale.
