# Supplementary material for: Antibiotic prescribing for lower UTI in elderly patients in primary care and risk of bloodstream infection: A cohort study using electronic health records in England
Source: PLoS Med. 2020 Sep 21;17(9):e1003336. doi: 10.1371/journal.pmed.1003336 (PMC7505443; doi:10.1371/journal.pmed.1003336)
Supplement: S3 Table — BSI, bloodstream infection; UTI, urinary tract infection. (DOCX) [file pmed.1003336.s004.docx]

**S3 Table -** Generalized estimating equation models of the association between immediate antibiotic prescribing for UTI and BSI in men. BSI, bloodstream infection; UTI, urinary tract infection.

|  |  |  |  |  |  |
| --- | --- | --- | --- | --- | --- |
|  | **Univariable analysis** | |  | **Multivariable analysis*** | |
| **Patient characteristics** | OR (95% CI) | p-value |  | aOR (95% CI) | p-value |
|  |  |  |  |  |  |
|  |  |  |  |  |  |
| **No antibiotic** | 0.93 (0.75-1.14) | 0.481 |  | 0.98 (0.79-1.21) | 0.845 |
|  |  |  |  |  |  |
| **Age** (continuous; per 5 years) | 1.27 (1.20-1.33) | <0.001 |  | 1.18 (1.11-1.25) | <0.001 |
| **IMD**  Q1 (least deprived) | 1 |  |  | 1 |  |
| Q2 | 1.09 (0.84-1.40) | 0.529 |  | 1.05 (0.82-1.36) | 0.685 |
| Q3 | 1.02 (0.78-1.32) | 0.908 |  | 0.98 (0.75-1.27) | 0.853 |
| Q4 | 1.26 (0.96-1.65) | 0.101 |  | 1.17 (0.89-1.55) | 0.258 |
| Q5 (most deprived) | 1.61 (1.21-2.13) | <0.001 |  | 1.40 (1.05-1.86) | 0.022 |
| **Region**  South of England | 1 |  |  | 1 |  |
| London | 1.08 (0.79-1.49) | 0.621 |  | 1.04 (0.76-1.44) | 0.789 |
| Midlands and east of England | 1.23 (1.00-1.52) | 0.054 |  | 1.16 (0.94-1.43) | 0.178 |
| North of England and Yorkshire | 1.45 (1.16-1.82) | 0.001 |  | 1.29 (1.02-1.62) | 0.030 |
| **NHS financial year**  2007/08 | 1 |  |  | 1 |  |
| 2008/09 | 0.89 (0.62-1.27) | 0.523 |  | 0.87 (0.60-1.24) | 0.435 |
| 2009/10 | 0.81 (0.56-1.16) | 0.250 |  | 0.76 (0.52-1.10) | 0.150 |
| 2010/11 | 0.87 (0.61-1.24) | 0.441 |  | 0.83 (0.58-1.20) | 0.322 |
| 2011/12 | 0.81 (0.57-1.15) | 0.231 |  | 0.79 (0.55-1.13) | 0.197 |
| 2012/13 | 0.83 (0.59-1.17) | 0.285 |  | 0.81 (0.57-1.15) | 0.242 |
| 2013/14 | 1.13 (0.82-1.56) | 0.462 |  | 1.15 (0.82-1.61) | 0.410 |
| 2014/15 | 1.37 (0.98-1.91) | 0.067 |  | 1.41 (1.00-1.99) | 0.053 |
| **CCI** (continuous) ^†^ | 1.56 (1.40-1.74) | <0.001 |  | 1.27 (1.13-1.44) | <0.001 |
| **Smoking status**  Non-smoker | 1 |  |  | 1 |  |
| Ex-smoker | 1.06 (0.89-1.27) | 0.510 |  | 0.98 (0.82-1.18) | 0.836 |
| Smoker | 1.02 (0.74-1.42) | 0.886 |  | 1.09 (0.78-1.52) | 0.612 |
| **Hospital stays**  Discharged from hospital in prior 7 days | 1.94 (1.43-2.65) | <0.001 |  | 1.45 (0.98-2.15) | 0.065 |
| Discharged from hospital in prior 30 days | 1.68 (1.35-2.08) | <0.001 |  | 1.09 (0.82-1.47) | 0.542 |
| Number of days spent in hospital  in prior year^†^ | 1.18 (1.15-1.21) | <0.001 |  | 1.09 (1.04-1.14) | <0.001 |
| Number of admissions in prior year^†^ | 1.89 (1.68-2.12) | <0.001 |  | 1.14 (0.91-1.42) | 0.265 |
| **A&E attendances**  A&E attendance in prior 30 days | 1.77 (1.33-2.37) | <0.001 |  | 1.13 (0.79-1.61) | 0.505 |
| Number of attendances in prior year^†^ | 1.56 (1.41-1.74) | <0.001 |  | 1.04 (0.87-1.24) | 0.657 |
| **Antibiotic in prior 30 days** | 1.50 (1.24-1.82) | <0.001 |  | 1.30 (1.06-1.58) | 0.010 |
| **Index event was home visit** | 3.13 (2.46-3.99) | <0.001 |  | 2.17 (1.67-2.82) | <0.001 |
|  |  |  |  |  |  |

A&E, accident and emergency; aOR, adjusted odds ratio; CCI, Charlson Comorbidity Index; IMD, Index of Multiple Deprivation 2015; NHS, UK National Health Service; OR, crude odds ratio; Q1–Q5, quintiles 1–5; UTI, urinary tract infection; 95% CI, 95% confidence interval.

* adjusted for all other variables included in the table

^†^ Transformed using the square root before input into the model. Effect sizes represent the relative change in odds (OR) *per 1 unit increase in the square root*, that is when the risk factor increases from 0 to 1, from 1 to 4, from 4 to 9, etc. on the original scale.
