# Supplementary material for: Antibiotic prescribing for lower UTI in elderly patients in primary care and risk of bloodstream infection: A cohort study using electronic health records in England
Source: PLoS Med. 2020 Sep 21;17(9):e1003336. doi: 10.1371/journal.pmed.1003336 (PMC7505443; doi:10.1371/journal.pmed.1003336)
Supplement: S4 Table — UTI, urinary tract infection. (DOCX) [file pmed.1003336.s005.docx]

**S4 Table -** Generalized estimating equation models of the association between immediate antibiotic prescribing for UTI and all-cause mortality within 60 days. UTI, urinary tract infection.

|  |  |  |  |  |  |
| --- | --- | --- | --- | --- | --- |
|  | **Univariable analysis** | |  | **Multivariable analysis*** | |
| **Patient characteristics** | OR (95% CI) | p-value |  | aOR (95% CI) | p-value |
|  |  |  |  |  |  |
|  |  |  |  |  |  |
| **No antibiotic** | 1.58 (1.47-1.69) | <0.001 |  | 1.17 (1.09-1.26) | <0.001 |
|  |  |  |  |  |  |
| **Age** (continuous; per 5 years) | 1.72 (1.70-1.75) | <0.001 |  | 1.62 (1.59-1.65) | <0.001 |
| **Female gender** | 0.49 (0.47-0.52) | <0.001 |  | 0.57 (0.53-0.60) | <0.001 |
| **IMD**  Q1 (least deprived) | 1 |  |  | 1 |  |
| Q2 | 1.16 (1.08-1.26) | <0.001 |  | 1.14 (1.05-1.24) | 0.001 |
| Q3 | 1.27 (1.17-1.37) | <0.001 |  | 1.18 (1.09-1.29) | <0.001 |
| Q4 | 1.20 (1.10-1.31) | <0.001 |  | 1.15 (1.05-1.26) | 0.002 |
| Q5 (most deprived) | 1.24 (1.13-1.36) | <0.001 |  | 1.12 (1.01-1.23) | 0.031 |
| **Region**  South of England | 1 |  |  | 1 |  |
| London | 0.81 (0.73-0.90) | <0.001 |  | 0.82 (0.73-0.91) | <0.001 |
| Midlands and east of England | 1.04 (0.98-1.11) | 0.219 |  | 0.99 (0.93-1.06) | 0.764 |
| North of England and Yorkshire | 1.02 (0.95-1.10) | 0.500 |  | 0.99 (0.92-1.06) | 0.728 |
| **NHS financial year**  2007/08 | 1 |  |  | 1 |  |
| 2008/09 | 0.98 (0.88-1.08) | 0.651 |  | 0.97 (0.87-1.08) | 0.592 |
| 2009/10 | 0.92 (0.83-1.02) | 0.099 |  | 0.88 (0.79-0.98) | 0.016 |
| 2010/11 | 0.91 (0.82-1.01) | 0.079 |  | 0.86 (0.77-0.96) | 0.008 |
| 2011/12 | 0.88 (0.80-0.98) | 0.018 |  | 0.81 (0.73-0.91) | <0.001 |
| 2012/13 | 0.96 (0.87-1.06) | 0.428 |  | 0.89 (0.80-0.99) | 0.038 |
| 2013/14 | 0.84 (0.75-0.93) | 0.001 |  | 0.78 (0.69-0.87) | <0.001 |
| 2014/15 | 1.02 (0.91-1.14) | 0.747 |  | 0.92 (0.82-1.04) | 0.190 |
| **CCI** (continuous) ^†^ | 2.09 (2.02-2.17) | <0.001 |  | 1.52 (1.46-1.59) | <0.001 |
| **Smoking status**  Non-smoker | 1 |  |  | 1 |  |
| Ex-smoker | 0.99 (0.94-1.05) | 0.779 |  | 0.88 (0.83-0.94) | <0.001 |
| Smoker | 0.94 (0.85-1.05) | 0.283 |  | 1.35 (1.20-1.51) | <0.001 |
| **Hospital stays**  Discharged from hospital in prior 7 days | 2.49 (2.22-2.80) | <0.001 |  | 1.04 (0.90-1.21) | 0.565 |
| Discharged from hospital in prior 30 days | 2.55 (2.38-2.74) | <0.001 |  | 1.43 (1.29-1.58) | <0.001 |
| Number of days spent in hospital  in prior year^†^ | 1.26 (1.25-1.27) | <0.001 |  | 1.09 (1.08-1.11) | <0.001 |
| Number of admissions in prior year^†^ | 2.65 (2.56-2.75) | <0.001 |  | 1.40 (1.30-1.51) | <0.001 |
| **A&E attendances**  A&E attendance in prior 30 days | 2.62 (2.39-2.88) | <0.001 |  | 1.30 (1.16-1.47) | <0.001 |
| Number of attendances in prior year^†^ | 1.87 (1.80-1.93) | <0.001 |  | 0.93 (0.87-0.98) | 0.010 |
| **Antibiotic in prior 30 days** | 1.55 (1.46-1.64) | <0.001 |  | 1.25 (1.17-1.33) | <0.001 |
| **Index event was home visit** | 3.82 (3.26-4.46) | <0.001 |  | 2.30 (2.13-2.49) | <0.001 |
|  |  |  |  |  |  |

A&E, accident and emergency; aOR, adjusted odds ratio; CCI, Charlson Comorbidity Index; IMD, Index of Multiple Deprivation 2015; NHS, UK National Health Service; OR, crude odds ratio; Q1–Q5, quintiles 1–5; UTI, urinary tract infection; 95% CI, 95% confidence interval.

* adjusted for all other variables included in the table

^†^ Transformed using the square root before input into the model. Effect sizes represent the relative change in odds (OR) *per 1 unit increase in the square root*, that is when the risk factor increases from 0 to 1, from 1 to 4, from 4 to 9, etc. on the original scale.
