# Supplementary material for: Antibiotic prescribing for lower UTI in elderly patients in primary care and risk of bloodstream infection: A cohort study using electronic health records in England
Source: PLoS Med. 2020 Sep 21;17(9):e1003336. doi: 10.1371/journal.pmed.1003336 (PMC7505443; doi:10.1371/journal.pmed.1003336)
Supplement: S5 Table — BSI, bloodstream infection; UTI, urinary tract infection. (DOCX) [file pmed.1003336.s006.docx]

**S5 Table -** Generalized estimating equation models of the association between immediate antibiotic prescribing for UTI and hospitalizations without evidence of UTI or BSI within 60 days. BSI, bloodstream infection; UTI, urinary tract infection.

|  |  |  |  |  |  |
| --- | --- | --- | --- | --- | --- |
|  | **Univariable analysis** | |  | **Multivariable analysis*** | |
| **Patient characteristics** | OR (95% CI) | p-value |  | aOR (95% CI) | p-value |
|  |  |  |  |  |  |
|  |  |  |  |  |  |
| **No antibiotic** | 1.41 (1.35-1.47) | <0.001 |  | 1.20 (1.15-1.25) | <0.001 |
|  |  |  |  |  |  |
| **Age** (continuous; per 5 years) | 1.27 (1.26-1.28) | <0.001 |  | 1.18 (1.17-1.19) | <0.001 |
| **Female gender** | 0.56 (0.54-0.58) | <0.001 |  | 0.70 (0.68-0.73) | <0.001 |
| **IMD**  Q1 (least deprived) | 1 |  |  | 1 |  |
| Q2 | 1.08 (1.03-1.14) | 0.001 |  | 1.03 (0.98-1.09) | 0.178 |
| Q3 | 1.17 (1.12-1.23) | <0.001 |  | 1.08 (1.03-1.14) | 0.001 |
| Q4 | 1.27 (1.20-1.34) | <0.001 |  | 1.15 (1.09-1.21) | <0.001 |
| Q5 (most deprived) | 1.48 (1.40-1.57) | <0.001 |  | 1.21 (1.14-1.28) | <0.001 |
| **Region**  South of England | 1 |  |  | 1 |  |
| London | 1.17 (1.10-1.24) | <0.001 |  | 1.07 (1.01-1.13) | 0.027 |
| Midlands and east of England | 1.15 (1.10-1.20) | <0.001 |  | 1.08 (1.04-1.12) | <0.001 |
| North of England and Yorkshire | 1.28 (1.23-1.34) | <0.001 |  | 1.14 (1.09-1.19) | <0.001 |
| **NHS financial year**  2007/08 | 1 |  |  | 1 |  |
| 2008/09 | 1.13 (1.06-1.20) | <0.001 |  | 1.06 (1.00-1.13) | 0.070 |
| 2009/10 | 1.13 (1.06-1.20) | <0.001 |  | 1.02 (0.95-1.08) | 0.618 |
| 2010/11 | 1.10 (1.03-1.17) | 0.003 |  | 0.97 (0.91-1.03) | 0.357 |
| 2011/12 | 1.06 (0.99-1.12) | 0.091 |  | 0.91 (0.85-0.97) | 0.005 |
| 2012/13 | 1.03 (0.97-1.09) | 0.378 |  | 0.88 (0.82-0.94) | <0.001 |
| 2013/14 | 1.03 (0.97-1.10) | 0.289 |  | 0.89 (0.83-0.95) | <0.001 |
| 2014/15 | 1.09 (1.02-1.17) | 0.014 |  | 0.93 (0.86-1.00) | 0.038 |
| **CCI** (continuous) ^†^ | 1.72 (1.68-1.76) | <0.001 |  | 1.34 (1.31-1.37) | <0.001 |
| **Smoking status**  Non-smoker | 1 |  |  | 1 |  |
| Ex-smoker | 1.25 (1.21-1.30) | <0.001 |  | 1.07 (1.03-1.11) | <0.001 |
| Smoker | 1.34 (1.26-1.42) | <0.001 |  | 1.39 (1.31-1.48) | <0.001 |
| **Hospital stays**  Discharged from hospital in prior 7 days | 2.68 (2.50-2.88) | <0.001 |  | 1.16 (1.06-1.27) | 0.001 |
| Discharged from hospital in prior 30 days | 2.57 (2.46-2.69) | <0.001 |  | 1.38 (1.30-1.47) | <0.001 |
| Number of days spent in hospital  in prior year^†^ | 1.23 (1.22-1.24) | <0.001 |  | 1.02 (1.01-1.04) | <0.001 |
| Number of admissions in prior year^†^ | 2.66 (2.60-2.73) | <0.001 |  | 1.61 (1.53-1.68) | <0.001 |
| **A&E attendances**  A&E attendance in prior 30 days | 2.79 (2.64-2.95) | <0.001 |  | 1.21 (1.13-1.30) | <0.001 |
| Number of attendances in prior year^†^ | 2.07 (2.02-2.11) | <0.001 |  | 1.21 (1.17-1.25) | <0.001 |
| **Antibiotic in prior 30 days** | 1.33 (1.28-1.38) | <0.001 |  | 1.11 (1.07-1.15) | <0.001 |
| **Index event was home visit** | 3.82 (3.26-4.46) | <0.001 |  | 1.59 (1.50-1.69) | <0.001 |
|  |  |  |  |  |  |

A&E, accident and emergency; aOR, adjusted odds ratio; CCI, Charlson Comorbidity Index; IMD, Index of Multiple Deprivation 2015; NHS, UK National Health Service; OR, crude odds ratio; Q1–Q5, quintiles 1–5; UTI, urinary tract infection; 95% CI, 95% confidence interval.

* adjusted for all other variables included in the table

^†^ Transformed using the square root before input into the model. Effect sizes represent the relative change in odds (OR) *per 1 unit increase in the square root*, that is when the risk factor increases from 0 to 1, from 1 to 4, from 4 to 9, etc. on the original scale.
