# Supplementary material for: Antibiotic prescribing for lower UTI in elderly patients in primary care and risk of bloodstream infection: A cohort study using electronic health records in England
Source: PLoS Med. 2020 Sep 21;17(9):e1003336. doi: 10.1371/journal.pmed.1003336 (PMC7505443; doi:10.1371/journal.pmed.1003336)
Supplement: S6 Table — BSI, bloodstream infection; UTI, urinary tract infection. (DOCX) [file pmed.1003336.s007.docx]

**S6 Table** - Generalized estimating equation models of the association between immediate antibiotic prescribing for UTI and BSI within 60 days for a patient’s first episode. BSI, bloodstream infection; UTI, urinary tract infection.

|  |  |  |  |  |  |
| --- | --- | --- | --- | --- | --- |
|  | **Univariable analysis** | |  | **Multivariable analysis*** | |
| **Patient characteristics** | OR (95% CI) | p-value |  | aOR (95% CI) | p-value |
|  |  |  |  |  |  |
|  |  |  |  |  |  |
| **No antibiotic** | 1.32 (1.09-1.60) | 0.004 |  | 0.97 (0.80-1.19) | 0.774 |
|  |  |  |  |  |  |
| **Age** (continuous; per 5 years) | 1.31 (1.26-1.36) | <0.001 |  | 1.23 (1.17-1.29) | <0.001 |
| **Female gender** | 0.43 (0.37-0.50) | <0.001 |  | 0.51 (0.43-0.60) | <0.001 |
| **IMD**  Q1 (least deprived) | 1 |  |  | 1 |  |
| Q2 | 1.51 (1.20-1.90) | <0.001 |  | 1.45 (1.16-1.83) | 0.001 |
| Q3 | 1.38 (1.09-1.75) | 0.008 |  | 1.30 (1.03-1.66) | 0.030 |
| Q4 | 1.60 (1.25-2.04) | <0.001 |  | 1.50 (1.17-1.92) | 0.001 |
| Q5 (most deprived) | 1.91 (1.48-2.45) | <0.001 |  | 1.62 (1.25-2.11) | <0.001 |
| **Region**  South of England | 1 |  |  | 1 |  |
| London | 0.98 (0.75-1.29) | 0.881 |  | 0.93 (0.71-1.23) | 0.619 |
| Midlands and east of England | 1.18 (0.98-1.41) | 0.073 |  | 1.13 (0.94-1.35) | 0.205 |
| North of England and Yorkshire | 1.28 (1.06-1.55) | 0.011 |  | 1.17 (0.96-1.43) | 0.125 |
| **NHS financial year**  2007/08 | 1 |  |  | 1 |  |
| 2008/09 | 1.09 (0.84-1.41) | 0.529 |  | 1.08 (0.83-1.40) | 0.579 |
| 2009/10 | 0.83 (0.62-1.11) | 0.206 |  | 0.80 (0.59-1.07) | 0.135 |
| 2010/11 | 1.13 (0.86-1.49) | 0.363 |  | 1.08 (0.82-1.43) | 0.588 |
| 2011/12 | 0.98 (0.73-1.31) | 0.886 |  | 0.94 (0.69-1.27) | 0.672 |
| 2012/13 | 1.16 (0.88-1.52) | 0.293 |  | 1.11 (0.83-1.47) | 0.488 |
| 2013/14 | 1.31 (0.99-1.72) | 0.05 |  | 1.28 (0.96-1.71) | 0.091 |
| 2014/15 | 1.72 (1.29-2.28) | <0.001 |  | 1.68 (1.25-2.27) | <0.001 |
| **CCI** (continuous) ^†^ | 1.77 (1.61-1.94) | <0.001 |  | 1.33 (1.20-1.47) | <0.001 |
| **Smoking status**  Non-smoker | 1 |  |  | 1 |  |
| Ex-smoker | 1.18 (1.01-1.38) | 0.043 |  | 0.93 (0.79-1.09) | 0.366 |
| Smoker | 1.38 (1.07-1.78) | 0.012 |  | 1.38 (1.06-1.80) | 0.015 |
| **Hospital stays**  Discharged from hospital in prior 7 days | 3.27 (2.50-4.27) | <0.001 |  | 1.67 (1.17-2.38) | 0.005 |
| Discharged from hospital in prior 30 days | 2.52 (2.08-3.05) | <0.001 |  | 1.16 (0.89-1.52) | 0.270 |
| Number of days spent in hospital  in prior year^†^ | 1.21 (1.19-1.24) | <0.001 |  | 1.09 (1.04-1.13) | <0.001 |
| Number of admissions in prior year^†^ | 2.28 (2.06-2.51) | <0.001 |  | 1.29 (1.05-1.58) | 0.013 |
| **A&E attendances**  A&E attendance in prior 30 days | 2.27 (1.75-2.93) | <0.001 |  | 1.18 (0.85-1.65) | 0.318 |
| Number of attendances in prior year^†^ | 1.70 (1.55-1.86) | <0.001 |  | 0.92 (0.78-1.08) | 0.315 |
| **Antibiotic in prior 30 days** | 1.42 (1.19-1.69) | <0.001 |  | 1.21 (1.01-1.45) | 0.038 |
| **Index event was home visit** | 3.84 (3.13-4.70) | <0.001 |  | 2.28 (1.83-2.85) | <0.001 |
|  |  |  |  |  |  |

A&E, accident and emergency; aOR, adjusted odds ratio; CCI, Charlson Comorbidity Index; IMD, Index of Multiple Deprivation 2015; NHS, UK National Health Service; OR, crude odds ratio; Q1–Q5, quintiles 1–5; UTI, urinary tract infection; 95% CI, 95% confidence interval.

* adjusted for all other variables included in the table

^†^ Transformed using the square root before input into the model. Effect sizes represent the relative change in odds (OR) *per 1 unit increase in the square root*, that is when the risk factor increases from 0 to 1, from 1 to 4, from 4 to 9, etc. on the original scale.
