# Supplementary material for: Antibiotic prescribing for lower UTI in elderly patients in primary care and risk of bloodstream infection: A cohort study using electronic health records in England
Source: PLoS Med. 2020 Sep 21;17(9):e1003336. doi: 10.1371/journal.pmed.1003336 (PMC7505443; doi:10.1371/journal.pmed.1003336)
Supplement: S7 Table — BSI, bloodstream infection; UTI, urinary tract infection. (DOCX) [file pmed.1003336.s008.docx]

**S7 Table** - Generalized estimating equation models of the association between immediate antibiotic prescribing for UTI and BSI within 30 days among all episodes. BSI, bloodstream infection; UTI, urinary tract infection.

|  |  |  |  |  |  |
| --- | --- | --- | --- | --- | --- |
|  | **Univariable analysis** | |  | **Multivariable analysis*** | |
| **Patient characteristics** | OR (95% CI) | p-value |  | aOR (95% CI) | p-value |
|  |  |  |  |  |  |
|  |  |  |  |  |  |
| **No antibiotic** | 1.71 (1.46-2.00) | <0.001 |  | 1.26 (1.07-1.48) | 0.006 |
|  |  |  |  |  |  |
| **Age** (continuous; per 5 years) | 1.31 (1.27-1.36) | <0.001 |  | 1.21 (1.16-1.26) | <0.001 |
| **Female gender** | 0.37 (0.32-0.42) | <0.001 |  | 0.46 (0.40-0.53) | <0.001 |
| **IMD**  Q1 (least deprived) | 1 |  |  | 1 |  |
| Q2 | 1.22 (1.01-1.47) | 0.041 |  | 1.18 (0.98-1.43) | 0.083 |
| Q3 | 1.13 (0.92-1.37) | 0.236 |  | 1.07 (0.87-1.30) | 0.525 |
| Q4 | 1.29 (1.05-1.59) | 0.016 |  | 1.21 (0.98-1.49) | 0.078 |
| Q5 (most deprived) | 1.49 (1.20-1.84) | <0.001 |  | 1.29 (1.03-1.61) | 0.027 |
| **Region**  South of England | 1 |  |  | 1 |  |
| London | 0.99 (0.78-1.26) | 0.923 |  | 0.93 (0.73-1.19) | 0.562 |
| Midlands and east of England | 1.20 (1.02-1.40) | 0.023 |  | 1.14 (0.97-1.33) | 0.107 |
| North of England and Yorkshire | 1.17 (0.99-1.39) | 0.066 |  | 1.08 (0.91-1.29) | 0.393 |
| **NHS financial year**  2007/08 | 1 |  |  | 1 |  |
| 2008/09 | 0.99 (0.76-1.30) | 0.939 |  | 0.97 (0.74-1.27) | 0.805 |
| 2009/10 | 0.83 (0.62-1.09) | 0.184 |  | 0.78 (0.59-1.04) | 0.087 |
| 2010/11 | 1.09 (0.84-1.42) | 0.518 |  | 1.01 (0.78-1.33) | 0.918 |
| 2011/12 | 0.94 (0.72-1.23) | 0.638 |  | 0.86 (0.65-1.13) | 0.282 |
| 2012/13 | 1.20 (0.93-1.55) | 0.166 |  | 1.09 (0.84-1.41) | 0.531 |
| 2013/14 | 1.38 (1.08-1.78) | 0.011 |  | 1.26 (0.97-1.63) | 0.079 |
| 2014/15 | 1.83 (1.42-2.35) | <0.001 |  | 1.65 (1.27-2.14) | <0.001 |
| **CCI** (continuous) ^†^ | 1.88 (1.73-2.05) | <0.001 |  | 1.40 (1.28-1.53) | <0.001 |
| **Smoking status**  Non-smoker | 1 |  |  | 1 |  |
| Ex-smoker | 1.16 (1.01-1.33) | 0.031 |  | 0.89 (0.77-1.02) | 0.096 |
| Smoker | 1.30 (1.03-1.65) | 0.027 |  | 1.31 (1.02-1.66) | 0.032 |
| **Hospital stays**  Discharged from hospital in prior 7 days | 3.48 (2.74-4.42) | <0.001 |  | 1.72 (1.26-2.35) | <0.001 |
| Discharged from hospital in prior 30 days | 2.54 (2.14-3.01) | <0.001 |  | 1.09 (0.86-1.39) | 0.471 |
| Number of days spent in hospital  in prior year^†^ | 1.23 (1.21-1.25) | <0.001 |  | 1.08 (1.04-1.11) | <0.001 |
| Number of admissions in prior year^†^ | 2.41 (2.22-2.62) | <0.001 |  | 1.34 (1.13-1.59) | <0.001 |
| **A&E attendances**  A&E attendance in prior 30 days | 2.68 (2.17-3.32) | <0.001 |  | 1.27 (0.97-1.66) | 0.085 |
| Number of attendances in prior year^†^ | 1.86 (1.73-2.00) | <0.001 |  | 1.02 (0.89-1.17) | 0.758 |
| **Antibiotic in prior 30 days** | 1.45 (1.26-1.67) | <0.001 |  | 1.19 (1.03-1.38) | 0.017 |
| **Index event was home visit** | 3.92 (3.29-4.68) | <0.001 |  | 2.23 (1.84-2.71) | <0.001 |
|  |  |  |  |  |  |

A&E, accident and emergency; aOR, adjusted odds ratio; CCI, Charlson Comorbidity Index; IMD, Index of Multiple Deprivation 2015; NHS, UK National Health Service; OR, crude odds ratio; Q1–Q5, quintiles 1–5; UTI, urinary tract infection; 95% CI, 95% confidence interval.

* adjusted for all other variables included in the table

^†^ Transformed using the square root before input into the model. Effect sizes represent the relative change in odds (OR) *per 1 unit increase in the square root*, that is when the risk factor increases from 0 to 1, from 1 to 4, from 4 to 9, etc. on the original scale.
