# Supplementary material for: Antibiotic prescribing for lower UTI in elderly patients in primary care and risk of bloodstream infection: A cohort study using electronic health records in England
Source: PLoS Med. 2020 Sep 21;17(9):e1003336. doi: 10.1371/journal.pmed.1003336 (PMC7505443; doi:10.1371/journal.pmed.1003336)
Supplement: S8 Table — In the case of matching 2 models were estimated, one using the general estimating equations used in the main analysis and a conditional logistic regression accounting for the matching procedure. (DOCX) [file pmed.1003336.s009.docx]

**S8 Table** – Propensity score analysis (parametric: logistic regression): results of the multivariate analysis are shown for both matching with up to 5 controls and inverse probability weighting. In the case of matching 2 models were estimated, one using the general estimating equations used in the main analysis and a conditional logistic regression accounting for the matching procedure.

|  |  |  |  |  |
| --- | --- | --- | --- | --- |
|  | **Matching** | |  | **Inverse probability weighting** |
|  | General estimating equation | Conditional logistic regression |  | General estimating equation |
| **Patient characteristics** | aOR (95% CI)* | aOR (95% CI)* |  | aOR (95% CI)* |
|  |  |  |  |  |
|  |  |  |  |  |
| **No antibiotic** | 1.11 (0.95-1.29) | 1.16 (0.98-1.36) |  | 1.26 (1.08-1.49) |
|  |  |  |  |  |
| **Age** (continuous; per 5 years) | 1.19 (1.14-1.24) | 1.20 (1.14-1.26) |  | 1.21 (1.17-1.26) |
| **Female gender** | 0.51 (0.44-0.58) | 0.45 (0.17-1.15) |  | 0.48 (0.43-0.55) |
| **IMD**  Q1 (least deprived) | 1 | 1 |  | 1 |
| Q2 | 1.24 (1.02-1.50) | 1.32 (1.06-1.64) |  | 1.21 (1.02-1.45) |
| Q3 | 1.20 (0.99-1.46) | 1.23 (0.98-1.55) |  | 1.22 (1.02-1.46) |
| Q4 | 1.30 (1.06-1.60) | 1.37 (1.07-1.74) |  | 1.30 (1.07-1.58) |
| Q5 (most deprived) | 1.54 (1.24-1.91) | 1.53 (1.19-1.97) |  | 1.44 (1.18-1.76) |
| **Region**  South of England | 1 | 1 |  | 1 |
| London | 1.03 (0.82-1.29) | 0.98 (0.75-1.28) |  | 1.03 (0.83-1.27) |
| Midlands and east of England | 1.10 (0.94-1.28) | 1.09 (0.91-1.31) |  | 1.14 (0.99-1.32) |
| North of England and Yorkshire | 1.16 (0.98-1.37) | 1.22 (0.95-1.56) |  | 1.17 (1.00-1.37) |
| **NHS financial year**  2007/08 | 1 | 1 |  | 1 |
| 2008/09 | 0.97 (0.74-1.27) | 0.94 (0.68-1.28) |  | 1.02 (0.80-1.30) |
| 2009/10 | 0.79 (0.60-1.05) | 0.82 (0.59-1.14) |  | 0.83 (0.65-1.07) |
| 2010/11 | 0.98 (0.75-1.28) | 1.02 (0.75-1.38) |  | 0.97 (0.77-1.24) |
| 2011/12 | 0.97 (0.75-1.27) | 0.85 (0.62-1.17) |  | 0.96 (0.75-1.22) |
| 2012/13 | 1.08 (0.83-1.39) | 1.05 (0.75-1.46) |  | 1.04 (0.82-1.32) |
| 2013/14 | 1.25 (0.97-1.60) | 1.08 (0.74-1.56) |  | 1.21 (0.95-1.52) |
| 2014/15 | 1.56 (1.20-2.02) | 1.44 (0.95-2.16) |  | 1.57 (1.24-1.99) |
| **CCI** (continuous) ^†^ | 1.38 (1.27-1.51) | 1.42 (1.28-1.59) |  | 1.42 (1.31-1.55) |
| **Smoking status**  Non-smoker | 1 | 1 |  | 1 |
| Ex-smoker | 0.98 (0.85-1.12) | 0.97 (0.83-1.14) |  | 0.94 (0.83-1.07) |
| Smoker | 1.21 (0.95-1.54) | 1.23 (0.93-1.64) |  | 1.18 (0.94-1.49) |
| **Hospital stays**  Discharged from hospital in prior 7 days | 1.31 (0.96-1.79) | 1.60 (0.99-2.59) |  | 1.36 (1.01-1.84) |
| Discharged from hospital in prior 30 days | 1.24 (0.98-1.56) | 1.31 (0.90-1.91) |  | 1.19 (0.96-1.48) |
| Number of days spent in hospital  in prior year^†^ | 1.09 (1.05-1.12) | 1.07 (1.03-1.12) |  | 1.08 (1.04-1.11) |
| Number of admissions in prior year^†^ | 1.24 (1.05-1.47) | 1.22 (1.00-1.49) |  | 1.29 (1.09-1.53) |
| **A&E attendances**  A&E attendance in prior 30 days | 1.15 (0.89-1.51) | 1.01 (0.60-1.71) |  | 1.19 (0.92-1.55) |
| Number of attendances in prior year^†^ | 0.97 (0.84-1.10) | 1.02 (0.88-1.20) |  | 0.98 (0.86-1.11) |
| **Antibiotic in prior 30 days** | 1.25 (1.09-1.43) | 1.17 (0.70-1.95) |  | 1.26 (1.10-1.44) |
| **Index event was home visit** | 2.23 (1.87-2.67) | 2.38 (0.95-5.98) |  | 2.23 (1.86-2.68) |
|  |  |  |  |  |

A&E, accident and emergency; aOR, adjusted odds ratio; CCI, Charlson Comorbidity Index; IMD, Index of Multiple Deprivation 2015; NHS, UK National Health Service; Q1–Q5, quintiles 1–5; 95% CI, 95% confidence interval.

* adjusted for all other variables included in the table

^†^ Transformed using the square root before input into the model. Effect sizes represent the relative change in odds (OR) *per 1 unit increase in the square root*, that is when the risk factor increases from 0 to 1, from 1 to 4, from 4 to 9, etc. on the original scale.
