# Supplementary material for: Antibiotic prescribing for lower UTI in elderly patients in primary care and risk of bloodstream infection: A cohort study using electronic health records in England
Source: PLoS Med. 2020 Sep 21;17(9):e1003336. doi: 10.1371/journal.pmed.1003336 (PMC7505443; doi:10.1371/journal.pmed.1003336)
Supplement: S1 Protocol — CPRD, Clinical Practice Research Datalink; HES, Hospital Episode Statistics; ONS, Office for National Statistics. (DOC) [file pmed.1003336.s011.doc]

**PROTOCOL INFORMATION REQUIRED**

The following sections below **must** be included in the CPRD ISAC research protocol. Please refer to the guidance on ‘***Contents of CPRD ISAC Research Protocols***’ ([www.cprd.com/isac](http://www.cprd.com/isac)) for more information on how to complete the sections below. Pages should be numbered. All abbreviations must be defined on first use.

| **Applicants must complete all sections listed below**  **Sections which do not apply should be completed as ‘*Not Applicable’*** |
| --- |
| 1. **Study Title^§^**   **^§^***Please note:* *This information will be published on CPRD’s website as part of its transparency policy*  The use and protective effect of antibiotics against complications of infection in patients in primary care: a cohort study using linked data from CPRD, HES and ONS |
| 1. **Lay Summary (Max. 200 words)^§^**   **^§^***Please note:* *This information will be published on CPRD’s website as part of its transparency policy*  We all rely on antibiotics to treat infections, but our supply of antibiotics that work is running out. Some bacteria that cause infections have become highly resistant to antibiotics. This “antibiotic resistance” is much more likely to happen if we use antibiotics too often when we don’t really need them. In the NHS, three-quarters of all antibiotics are prescribed by general practitioners (GPs). Often antibiotics are the right treatment for patients but sometimes patients are prescribed antibiotics for viral infections like coughs and colds, where antibiotics don’t work. Some patients receive lots of antibiotics, others get them very rarely. Some GPs seem to prescribe antibiotics more often than others. The aim of this study is to use anonymous GP medical records (so individual patients can’t be identified) to find out more about when and why antibiotics are prescribed in General Practice. We want to understand why some patients get antibiotics more often than others and when patients really need an antibiotic. We will use our work to develop computer simulations to help GPs decide when to prescribe antibiotics. By reducing the number of times that antibiotics are prescribed unnecessarily we will help to keep our current antibiotics working for longer. |
| 1. **Technical Summary (Max. 200 words**)**^§^**   **^§^***Please note:* *This information will be published on CPRD’s website as part of its transparency policy*  Antibiotic overuse drives antimicrobial resistance. In primary care rates of antibiotic prescribing vary widely, associated with characteristics of the patient and GP prescribing behaviour.  The aim of this study is to investigate whether analyses of patient level characteristics can guide antibiotic prescribing decisions in primary care.  Using data from patients who contributed to CPRD between April 2007 and December 2015, we will describe the diagnosis, antibiotic treatment and clinical outcomes of patients with common infection syndromes. These analyses will be stratified by patient characteristics including: age, gender, social deprivation, selected co-morbidities, obesity and smoking.  In a series of cohort studies of patients with common infection syndromes, we will: a) estimate the rate of adverse outcomes comparing those who were treated with antibiotics to those who were not, using Poisson regression and b) calculate the number needed to treat to avoid one infection related adverse outcome using multi-level logistic regression, taking account of patient’s vulnerability through the use of propensity scores. These analyses will be stratified by each of the patient characteristics listed above. This work will be synthesized through models that predict each patient’s risk of adverse outcomes, comparing the scenario of antibiotic treatment versus no antibiotic treatment. |
| 1. **Objectives, Specific Aims and Rationale**   *Objective*  To investigate the use of antibiotics and their protective effect against complications of infection to inform the development of risk prediction models to guide antibiotic treatment decisions for suspected infection in primary care.  *Aims*   1. Estimate the incidence of i) GP consultations for infection and ii) antibiotic prescriptions by: age-group, gender, SES, ethnicity, selected co-morbidities, obesity and smoking status 2. Describe antibiotic prescribing patterns linked to the reason for the prescription by: age-group, gender, SES, ethnicity, selected co-morbidities, obesity and smoking status 3. Estimate the incidence of adverse outcomes in the following 28 days including: hospital admission for infection; A&E admission; re-consultation for infection; re-consultation for antibiotic side-effects (rash, nausea, …) and death for patients with a GP consultation for i) respiratory tract infection ii) urinary tract iii) skin/soft tissue infection, comparing patients with each listed age/co-morbidity group (including smoking status and obesity) to non-comorbid patients 4. Estimate the number of patients needed to treat with antibiotics to avoid one infection-related adverse outcome, comparing the results for each age/co-morbidity group to non-comorbid patients 5. Develop risk prediction tools to support clinical decision making and communication with patients   *Rational*  We have previously shown that more than half of all antibiotic prescriptions in primary care are issued to less than 10% of patients, and that this 10% of patients have high levels of co-morbidity.^1^ Targeting such high-frequency users of antibiotics in antimicrobial stewardship programmes might provide opportunities for a more effective intervention approach. Underlying this hypothesis is the assumption that it might be safe and feasible to reduce prescribing in patients with the highest levels of antibiotic prescribing, many of whom have co-morbidity.  To investigate whether higher rates of antibiotic use are likely to be justified in higher risk patients compared to patients who lack these risk factors, we will undertake a series of studies to investigate which patients in terms of age, gender, SES, ethnicity, selected co-morbidities, obesity and smoking get the most antibiotics. We will determine which patients are most likely to receive antibiotics for which infections and relate this to national guidance on antibiotic prescribing. To estimate whether the protective effect of antibiotics differs according to the patient’s age and the presence of co-morbidity, obesity or their smoking status, we will estimate the risk of infection related adverse-outcomes, comparing patients who have been treated with antibiotics to those who have not been treated with antibiotics for each of these groups. We will use these estimates to calculate the number of patients who need to be treated with antibiotics to avoid one infection-related adverse outcome (NNT), accounting for age, gender, SES, ethnicity, co-morbidity, obesity and smoking status. This will inform the development and validation of individualised risk-prediction models to support clinical decision-making and patient communication around the need for antibiotic treatment for suspected infection in primary care. |
| 1. **Study Background**   Over the past 70 years antibiotics have revolutionised how we practice medicine, underpinning routine surgery through to cancer therapies and organ transplantation. Unfortunately, most antibiotics have a finite lifespan as drug resistant strains emerge; an inevitable consequence of bacterial evolution.  Resistance to antibiotics is largely driven by the inappropriate use and overuse of these drugs in humans and animals. Although the majority of highly drug resistant infections emerge in hospitals, 75% of antibiotics for human use are prescribed in primary care.^2^ The most common reasons for prescription of an antibiotic in primary care are for the treatment of respiratory tract, urinary tract, and skin and soft tissue infections. Despite guidelines on the management of these infections,^3^ large variations exist in the rates of antibiotic prescribing between individual practices in the UK^4–6^, with some practices prescribing at twice the rate compared to other sites.  Significant efforts have been made to understand the factors that influence the GP’s decision to prescribe an antibiotic.^7^ Examples of interventions that have been developed to reduce antibiotic use include symptom-based scoring systems or point-of-care diagnostics to improve diagnostic certainty.^8^ There have also been a range of initiatives to improve communication between the doctor and patient, to reduce unnecessary antibiotic use.^9^ The main tool currently used in primary care is the Treat Antibiotics Responsibly Guidance Education Tools (TARGET) toolkit, which was designed by Public Health England.^10^ It aims to influence prescribers’ and patients’ personal attitudes, social norms and perceived barriers to optimal antibiotic use.  To date, these interventions have targeted all patients who consult for an infection in primary care, despite the fact that some patients are never or rarely prescribed antibiotics, whereas others may have many courses of antibiotics in a single year. This patient-level variation in antibiotic prescribing is likely to be driven by a combination of demographic, behavioural and clinical factors including the presence of co-morbidity. For example, higher rates of antibiotic prescribing might be justified in patients with COPD for the treatment of bacterial infective exacerbations.^6^ By contrast, for conditions such as obesity, where there is also evidence that patients are more likely to be prescribed antibiotics,^1^ the clinical indication for increased antibiotic use is less clear.  We speculate that reductions in antibiotic resistance may require targeted public health interventions aimed at reducing antibiotic prescribing for patients who have the highest frequency antibiotic use. To test whether it might be safe and feasible to reduce antibiotic prescribing to patients with co-morbidity in clinical practice (who account for a substantial burden of total antibiotic use), we estimate the protective effect of antibiotic treatment against infection-related adverse outcomes, comparing effects in patients with co-morbidity to those in controls without co-morbidity. Based on this, there is then a need to develop tools enabling a better understanding of risk to support clinical decisions about which patients need antibiotics and communication with patients. |
| 1. **Study Type**   First we will **describe** the incidence of and relationships between antibiotic prescribing, GP consultation for infection and infection related adverse outcomes for all patients and for subgroups of patients based on risk factors including: age, gender, SES, co-morbidity, smoking status and obesity.  Next we will then **test the hypothesis** that the protective effect of antibiotics against adverse outcomes of infection differs between groups of patients with and without these risk factors.  Finally, we will use our results to inform the **methodological** development of risk prediction models to support clinical decisions around antibiotic treatment. |
| 1. **Study Design**   For this study, we will use a retrospective cohort study design, looking at patients with consultations for infection and/or antibiotic prescribing between 1^st^ April 2007 and 31^st^ December 2015. (for an exact definition see *K. Study population* and *Error: Reference source not found*)  In a first step (Objective 1), we will describe the incidence of GP consultations for infection and the incidence of antibiotic prescribing in the main study population. This will provide important context on the burden of infection and frequency of antibiotic use in primary care.  Within the main study population, we will then undertake a nested cross-sectional study looking at all antibiotic prescriptions in the main study population. For each prescription we will use Read codes on the day of the prescription to establish the reason for the prescription. (Objective 2) This will inform our understanding of why antibiotics are prescribed.  Next, we will undertake a series of cohort studies focused on specific infectious syndromes within the main population. Three nested cohorts will be built based on a GP consultation for a) respiratory tract infection, b) urinary tract infection and c) skin/soft tissue infection. For each cohort, we will estimate the rate of infection-related adverse outcomes within 28 days of the initial GP consultation for patients with and without antibiotic treatment. (Objective 3)  In order to determine the protective effects of antibiotics against these adverse outcomes, we will then estimate the Number Needed To Treat with antibiotics (NNT) to prevent one additional adverse outcome. In particular, we will compare the NNT for patients with and without co-morbidities of interest, obesity or smoking. (Objective 4).  Finally, we will synthesize out analyses in objectives 1-4 to develop a risk model for each cohort (respiratory, urinary and skin/soft tissue models). These models will support clinical decisions and patient communication around the need for antibiotic treatment for suspected infection (Objective 5).  Results across all steps will be stratified or adjusted by age, gender, SES, ethnicity, co-morbidity, obesity and smoking status. |
| 1. **Feasibility counts**   A study we conducted in The Health Improvement Network (THIN) using a comparable study population over the course of three years found that 30% of all patients had at least one prescription per year, with significantly higher rates of prescribing in co-morbid patients.^1^ The same study found 107,382 patients with diabetes, 79,056 patients with chronic kidney disease (CKD), 36,119 with chronic obstructive pulmonary disease (COPD), 216,379 patients with asthma, 395,825 patients who smoked and 358,930 patients who were obese. Numbers of prescription and co-morbidity in CPRD are expected to be proportionally similar to those obtained in THIN, i.e. we expect to find more patients given our longer study period of 10 years.  Figure 1 - Study design  **MAIN STUDY POPULATION – ALL PATIENTS IN DATASET MEETING STUDY INCLUSION CRITERIA**  **ESTIMATE INCIDENCE OF 1) GP CONSULTATION FOR INFECTION AND 2) ANTIBIOTIC PRESCRIPTION**  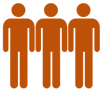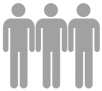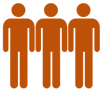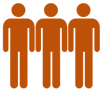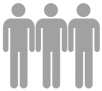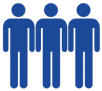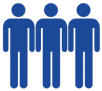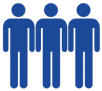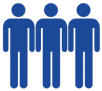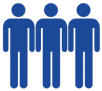  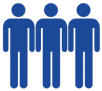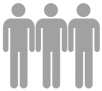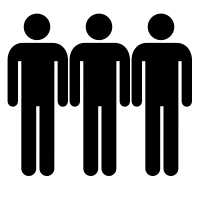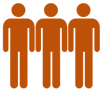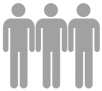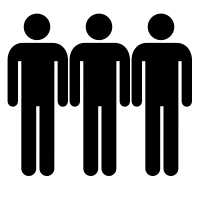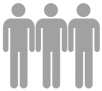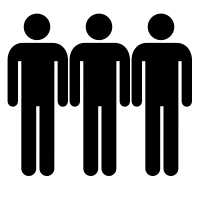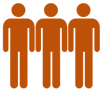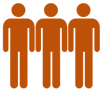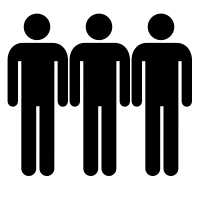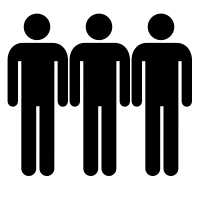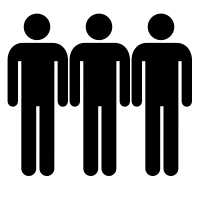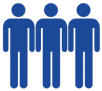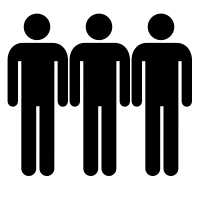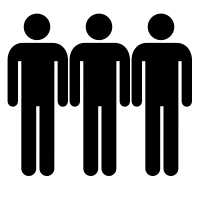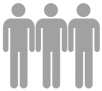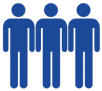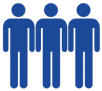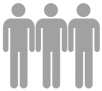  **NESTED CROSS-SECTIONAL STUDY SERIES OF NESTED COHORT STUDIES**  **Patterns of antibiotic prescribing linked to clinical indication for the prescription**  **Incidence of infection-related adverse events**  **Number needed to treat**  **Risk prediction models**  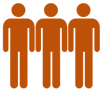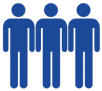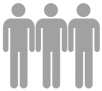 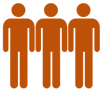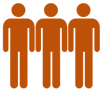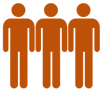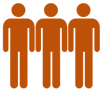  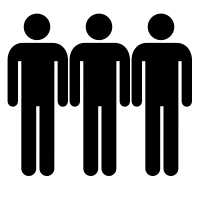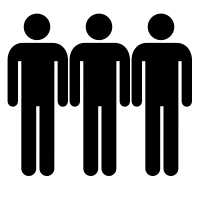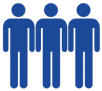 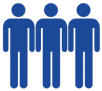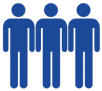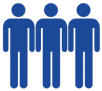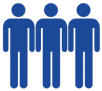  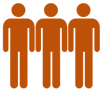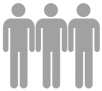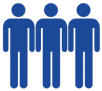 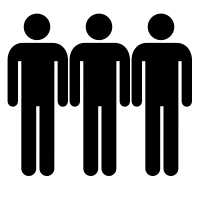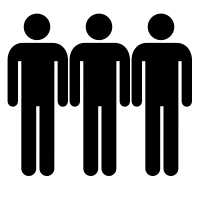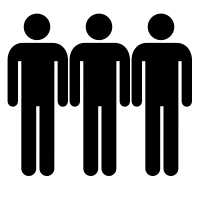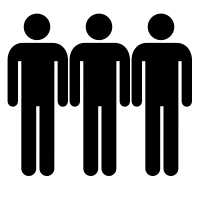  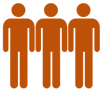 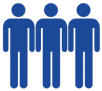 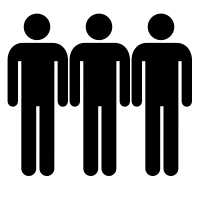 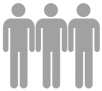  Respiratory tract Urinary tract Skin/soft tissue No recorded infection  Icons made by [Freepik](../Freepik) (https://www.flaticon.com/authors/freepik) from [www.flaticon.com](http://www.flaticon.com/) |
| 1. **Sample size considerations**   The main effects of interest in this study are the protective effect of antibiotic treatment (OR_AB_) and how this effect differs by co-morbidity (OR_CO_; i.e. the interaction between antibiotic treatment and co-morbidity).  Below, we derive sample sizes needed to detect these effects at 5% significance and 80% power (see Appendix Sample Size Calculation for detailed information on calculations and assumptions). We do so first for COPD patients with a respiratory infection based on previously published effect sizes. We will then relate these sample sizes to patients with other co-morbidities and with urinary or skin/soft tissue infections.  **Respiratory infections**  The power to detect any protective effects depends heavily on the risk of developing the outcome in question.  Past studies looked at a range of individual outcomes, including some extremely rare outcomes with risks as low as 0.04% (e.g. mastoiditis after URTI). They found average effect of antibiotics of OR_AB_ = 0.69.^11^ and a sample size of ***971,057*** is required to detect this effect in our study.^12^ To be able to further investigate interactions, i.e. is the protective effect of antibiotics against rare outcomes such as mastoiditis different in patients with COPD versus no COPD, a sample size of **approx. 14 million** patients would be required.^13^ This is beyond the scope of this study.  Within this study, we will not consider effect sizes in rare outcomes such as mastoiditis individually. Instead, we will look at composite outcomes relevant to patients who consulted for infections, i.e. outcomes such as re-consultations, hospitalisations, etc. (see M. Exposures, Health Outcomes§ and Covariates for details). The risk for these outcomes is much more likely to be around 2.4% previously found for pneumonia.^11^ For this risk, required sample sizes for the protective effect of antibiotic treatment (OR_AB_) and how this effect differs by co-morbidity (OR_CO_) reduce to ***16,512***and ***258,247***.  Assuming a total sample size of at least 3,355,353 found previously (based on only 162 practices^11^), this study will be powered to detect OR_AB_ and OR_CO_ as small as 0.975 and 0.83, i.e. we will have the power to detect a protective effect of antibiotics against adverse outcomes as small as 2.5% and an increase in effects of antibiotics by 17% points in co-morbid patients.  **Urinary tract and skin/ soft tissue infections**  There are roughly 1/5 as many cases of urinary tract infection and 2/5 as many cases of skin/soft tissue infections as compared to respiratory infections.^14^ This translates into 671,070 cases of urinary and 1,342,141 cases of skin/ soft tissue infection. No published figures on effect sizes in urinary or skin infections could be found. Assuming similar effect sizes as in respiratory infections, the numbers for both kinds of infection well exceed the minimum sample size of 258,247 needed.  **Other comorbidities**  The powers to detect the same effects for diabetes, CKD, asthma, smoking and obesity are expected to be the same or higher, given the higher prevalence for these conditions (H. Feasibility counts). |
| 1. **Data Linkage Required (if applicable):^§^**   **^§^***Please note that the data linkage/s requested in research protocols will be published by the CPRD as part of its transparency policy*  The study will make use of linked data, which will consist of linked CPRD, Office of National Statistics (ONS) mortality and Index of Multiple Deprivation (IMD) and Hospital Episode Statistics (HES) inpatient and A&E datasets. CPRD data will be obtained through the existing UCL institutional licence and up-to-date HES/ONS datasets will be obtained by making an appropriate request to CPRD.  Information in inpatient and A&E HES will be utilised to identify hospitalisation after presentation in primary care.  ONS data will be used as the source to a) determine death dates, and b) obtain and include social deprivation as a covariate in our models. |
| 1. **Study population**   The study period will be from 1^st^ April 2007 to 31^st^ December 2015.  We will perform all analyses in patients that were eligible for all linkages and fulfil the following:   1. registered at a participating English practice prior to the transfer of identifiers to the trusted third party for matching for at least a year before their entry into the study 2. their practice is deemed to be contributing ‘up-to-standard' (UTS) data at study start date 3. they had a valid identifier for linkage (NHS number for ONS mortality and HES data, postcode for IMD) 4. they had not opted out or dissented from CPRD or the linkage scheme 5. individual record is of ‘acceptable' research quality as verified by the CPRD.   **Cohort 1**: Individuals who were registered with a GP contributing to CPRD between 1^st^ April 2007 and 31^st^ December 2015 and who have a complete record on age, gender and IMD are eligible for inclusion in this study (see *P. Plans for addressing missing data*). Patients must have been registered with their GP for at least one year before entering the study with the exception of children aged less than one year who will be assumed to be born into the Practice. Patients can enter the study late or leave the study early in order to include patients who died within the study period (who might be inherently less healthy). Patients enter the study population on the latest of the following dates: continuous registration with GP for 12 months or 1^st^ April 2007. Where patients registered with their GP after the 1^st^ April 2007, patients enter the cohort after 12 months of continuous registration. Patients exit the cohort on the earliest of the following dates: the date the patient died, the date the patient left the practice, or 31^st^ December 2015.  Subsequent studies are nested within cohort 1 (see Error: Reference source not found):   1. Nested cross-sectional study: Patients within cohort 1 with at least one prescription of an antibiotic 2. Nested cohort studies of specific infection syndromes: Patients within cohort 1 with at least one GP consultation for a specific infection syndrome (respiratory, urinary, skin/soft tissue). For these cohorts, patients will enter the cohort on the day of the consultation for infection. Patients will leave the cohort on the earlier of: 28 days after initial consultation, death, transfer out of practice or 31st December 2015. Patients can enter and exit the cohort more than once. |
| 1. **Selection of comparison group(s) or controls**   In the cohort and cross-sectional studies outlined in objectives 1-4 we will compare rates of antibiotic prescribing and infection-related outcomes in different population sub-groups which will be defined by age-group, gender, SES, co-morbidity, smoking status and obesity.  To assess the number needed to treat with antibiotics to avoid one infection-related adverse event (objective 4) we will compare patients who have been treated with antibiotics (cases) to those who have not been treated with antibiotics (controls)  Objective 5 is limited to prediction and does not attempt comparison. |
| 1. **Exposures, Health Outcomes^§^ and Covariates**   **^§^***Please note:* *Summary information on health outcomes (as included on the ISAC application form above) will be published on CPRD’s website as part of its transparency policy*  *Exposure*  Exposures will include i) prescription of an antibiotic ii) GP consultation for suspected infection (see Appendices: Table 1 – Codelist for antibiotics, Table 2 – Codelist categorising indications for antibiotic use and Table 3 – Codelists to identify patients with suspected respiratory tract, urinary tract or skin/soft tissue infection)  *Health Outcomes*  The following outcomes (within 28 days of presentation in primary care) will be separately considered for analysis:   - Re-consultation for the same type of infection (CPRD; see Appendix Table 3) - Re-consultation for a side effect of antibiotic treatment (CPRD; see Appendix Table 4a –side effects of antibiotic treatment) - Complications of infection in primary or secondary care (CPRD and inpatient HES; see Appendices: Table 4b and 4c – complications of infection; depending on the indication) - Admission to Accident & Emergency (HES A&E) - All-cause hospitalisation (inpatient HES) - All-cause mortality (CPRD, HES and ONS)   We will undertake a sensitivity analysis to investigate the impact of excluding outcomes that occur on the same day as the exposure. The impact of different time windows will be explored, informed by a literature review on the natural history of the duration of different infections.  *Covariates*  We will include the following covariates:   - Age (classified as 0-4, 5-17, 18-44, 45-64, 65-84, 85-94 and 95+, years of age inclusive) - Sex (female and male) - Index of Multiple Deprivation (IMD; 1-5) - Co-morbidities (coded as indicator variables based on national guidance from Public Health England^3^ and feasibility^1^; see Appendix Table 5 for comorbidity code list)   - Diabetes   - Chronic Kidney Disease (CKD)   - Chronic Obstructive Pulmonary Disease (COPD)   - Asthma - Obesity - Smoking status^15^ - Season (months or quarters) and year - Ethnicity   We will undertake a sensitivity analysis of the impact of coding co-morbidities as binary indicator variable and as total number of co-morbidities. |
| 1. **Data/ Statistical Analysis**   *1. Estimate the incidence of i) GP consultations for infection and ii) antibiotic prescriptions*  For every patient in cohort 1, data on all consultations for infection and antibiotic prescriptions occurring within the study period will be extracted. We will estimate the incidence of GP consultations for infection and antibiotic prescriptions. This will be done for all consultations and prescriptions, and then separately for each category of infection and each class of antibiotics, based on chapter 5.1 of the British National Formulary (BNF) (see Appendix Tables 1 & 2). The denominator will be estimated as total person-time contributed by patients in the cohort during the study period. We will use Poisson regression to estimate the adjusted rate of consultation and prescription, taking account of the effect of age, gender, SES, ethnicity, selected co-morbidity, smoking and obesity. Random effects will be included to take account of clustering of observations by practice. Rates will be assessed for overdispersion by additionally fitting a negative binomial model and testing for alpha greater than zero via a likelihood-ratio test. If rates are found to be overdispersed, the negative binomial model will be used instead of a Poisson model. Adjusted rate ratios will be presented with 95%-confidence intervals.  *2. Describe antibiotic prescribing patterns linked to the reason for prescription*  The overall number of antibiotic prescribing episodes will be calculated for each patient individually, counting all recorded prescriptions between 1^st^ April 2007 and 31^st^ December 2015. For each episode, a clinical indication will be ascertained. Read codes for a diagnosis or symptom of infection recorded on the same day as the antibiotic prescription will be assumed to represent an indication for the prescription. Where more than one possible indication within the same type of infection is recorded on the day of prescription, the more granular code will be used. If multiple indications across different types of infection are recorded, the prescription will be labelled as having multiple indications. Conversely, if no infectious indication for a prescription was found, the prescription will be labelled as having no infectious indication and we will look for any non-infectious codes associated with the date of antibiotic prescription. The ten most common indications will be established and compared to the same numbers for the period from 1998-2001.^14^ This approach will be replicated for each class of antibiotics based on chapter 5.1 of the BNF. Patterns of repeat consultation or prescription during episodes of infection which may be prolonged will be investigated for each antibiotic prescription extracted in objective 1. We will define a new episode as a consultation or prescription recorded at least 28 days after any previous consultation for infection or antibiotic prescription. We will undertake sensitivity analyses to investigate the impact of using different time periods to define an episode. We will also examine the use of regular (prophylactic) antibiotic treatment.  Proportions and appropriate 95%-confidence intervals will be calculated and all results will be stratified by age-group, gender, SES, ethnicity, selected co-morbidity, obesity and smoking status. Descriptive data will be displayed graphically.  *3. Estimate the incidence of adverse outcomes in the following 28 days*  For each of the selected clinical infection syndromes in the nested cohort study, events in CPRD, HES and ONS will be searched for all adverse outcomes of interest (see *M.* Exposures, Health Outcomes§ and Covariates). Crude incidence rates of for each outcome will be calculated for patients who were treated (exposed) and not treated (unexposed) with an antibiotic. The denominator will be estimated as the total person-time contributed by patients at risk of each adverse outcome, taking account of the fact that this will be different for each outcome. For example, exposed and unexposed patients cannot consult General Practice if they have been admitted to hospital and should therefore not contribute to the denominator during this period when estimating rates of GP re-consultation in the 28 days following consultation for infection. A Poisson regression model will be used to estimate adjusted rates of adverse outcomes in each group, controlling for age, gender, socio-economic status, ethnicity, co-morbidity, obesity and smoking status and assessing for interaction between age and co-morbidity. Random effects will be included to take account of clustering of observations by practice. If the outcomes are overdispersed, negative binomial models will be used instead of Poisson models. Adjusted rate ratios will be presented with 95%-confidence intervals.  *4. Estimate the number of patients needed to treat with antibiotics to avoid one infection-related adverse outcome*  For each clinical infection syndrome-related adverse outcome identified in objective 3, the odds of that outcome in the 28 days following consultation for those treated with antibiotics (unexposed) and not treated with antibiotics (exposed) will be estimated using logistic regression. We will calculate adjusted odds ratios for the protective effect of antibiotics, adjusting for age, gender, SES, ethnicity, year/season, co-morbidity and the probability of receiving antibiotic treatment (see *O. Plan for addressing confounding*) and assessing for interactions with age and co-morbidity. We will use mixed models to adjust for clustering at a practice level and to take account of clustering of observations within a patient due to repeat episodes of infection. Where there is a significant effect of antibiotics between treated and untreated patients, the NNT or NNH to prevent one individual from developing an adverse outcome is calculated as *1/(absolute risk difference)*. A significant effect will be defined by a p-value < 0.05 for the coefficients of antibiotic treatment and its interactions. To avoid an arbitrary cut-off point, p-values close to 0.05 will also be assessed but discussed separately. The NNT will then be used to assess the clinical relevance of any significant differences found. Whilst the relative risk reduction (RRR) may be similar, the NNT may be different due to differing background risk. This will be explored.  *5. Develop risk prediction tools to support clinical decision making*  For each cohort (i.e. for each of the clinical syndromes of UTI, RTI and SSTI), we will develop a logistic regression risk model to predict the risk of adverse outcomes if patients are treated versus not treated with antibiotics. Models will be developed on a random sample of 80% of the observations within a cohort, reserving 20% of the data to estimate the model’s predictive performance.  A negative outcome will be defined as the occurrence of any of the following within 28 days following the index consultation: hospital admission for infection or complication; re-consultation for infection; A&E admission; death. Antibiotic treatment will be defined as a prescription of an antibiotic on the day of consultation for clinical syndrome. Risk factors will be selected based on the analyses outlined in objectives 1-4 and broadly grouped into: socio-demographic variables, co-morbidity and clinical presentation. Biologically plausible interactions between risk factors and age and between risk factors and co-morbidities will be examined.  Whereas age and sex will be forced into the model, other risk factors will be added in order of their significance in objective 4. Risk factors will be kept in the model if they improve the predictive performance of the model as defined by model discrimination and model calibration.^17^ For example, a risk factor will be used in the model if it improves discrimination and/or calibration. If a variable improves discrimination but reduces calibration, it will still be included in the model, but will be omitted if it increases calibration at the cost of discrimination.  Model discrimination, i.e. the model’s ability to correctly identify patients with and without an outcome, will be estimated using the concordance (c) statistic.^18^ The c statistic represents the probability that between two randomly chosen patients, one with the outcome and one without, the model assigns a higher score to the patient with the outcome. As a second measure, model calibration will be assessed using the Hosmer-Lemeshow test.^18^ We will additionally assess model calibration graphically for reasons for poor calibration by comparing the mean observed outcome in deciles of predicted risk. In this calibration plot, the intercept indicates the extent to which predictions are systematically too low or too high, while the slope reflects overfitting of the model.  For each model, we will estimate the internal validity of discrimination and calibration estimates using cross-validation with bootstrap subsampling based on 1000 iterations. In a secondary analysis, we will assess model performance in subgroups by age, sex and co-morbidity.  Finally, we will develop a simplified scoring system using the risk factors in the model,^17^ assigning the total score to a specific absolute risk of adverse outcomes in the 28 days following consultation, comparing outcomes if an individual is treated versus not treated with antibiotics. We will assess the predictive performance of the risk scores using the approaches outlined above. |
| 1. **Plan for addressing confounding**   In objectives 1-3 the aim is to describe rates and undertake descriptive analyses rather than to make inferences about the effect of antibiotic treatment, which is the purpose of objectives 4 and 5. For this reason the issue of confounding is only relevant to objectives 4 and 5.  Individuals who are at higher risk of complications will be more likely to be prescribed antibiotics by their GP (i.e. confounding by indication), introducing a systematic difference between those who are treated and not treated with antibiotics. In this scenario, untreated cases will be less likely to experience adverse events irrespective of their exposure to antibiotics, tending to underestimate the protective effect of antibiotics. We will use propensity score methods to attempt to adjust for this issue. These methods are increasingly used to mitigate the consequences of non-random allocation of treatment in observational studies by modelling the a priori probability of each patient to receive the treatment.  We will use the treatment propensity scores to perform an inverse probability of treatment weighting to account for baseline differences in treated and untreated participants. This will enable assessment of absolute risk and numbers needed to treat to prevent re-consultation and hospitalisation in different groups.^19^ Propensity score weighting in effect produces a synthetic sample in which the distribution of measured baseline covariates is independent of treatment assignment. Presentation and analyses of results are very similar to a randomized trial allowing assessment of absolute risk and numbers needed to treat in different groups.  In order to create appropriate propensity scores, we will attempt to capture indicators for severity of disease and patients’ risk of complication, such as prior hospitalisation and immunosuppression. Measures such as vital signs are usually poorly recorded which can hamper efforts to estimate disease severity. To overcome this issue we will use GP prescribing preference as an instrumental variables, as described by Davies et al 2013.^20^  Besides confounding by indication, a number of further factors might influence prescribing. The decision to prescribe an antibiotic is a complex process, depending on multiple characteristics on the patient and practice level. Patients’ demographic factors, patients’ attitude towards antibiotic prescribing, physicians’ workload, date of consultation and geographic location of the practice have been found to influence physicians’ prescribing decisions.^21^ The use of further instrumental variables may help to estimate the impact of these factors in our analysis and we will also control for differences in prescribing habits between practices through the application of random effects models taking account of potential correlation within multiple episodes of the same patient and between patients within the same practice.  Acknowledging that differences in practice prescribing do not necessarily constitute actually differing prescribing habits but might instead reflect underlying differences in coding, we will undertake an exploratory analysis to look at variation between practices graphically. Using a novel approach we will visualise variability between multiple data-generating sources (i.e. practices) that was specifically designed and tested for multitype, multivariate and multimodal biomedical data.^22,23^ Where outlying practices are identified, analysis will be re-run without those practices and results presented with the analysis outlined in *N. Data/ Statistical Analysis*. |
| 1. **Plans for addressing missing data**   Only records with information on age, gender and SES will be included in the analysis. For variables such as BMI, smoking and ethnicity, where missing data are likely, we will tabulate the patterns of missing data to identify plausible reasons for missing values. In the unlikely scenario that data are missing completely at random, we will undertake a complete case analysis. If the missing data depends on the outcome, which is more plausible, we will use multiple imputation. We will assess different assumptions about why data are missing and assess the impact missing data may have on the analysis. Importantly for this study, data on antibiotic prescriptions are particularly well-recorded because prescriptions are issued electronically. |
| 1. **Patient or user group involvement (if applicable)**   Not applicable |
| 1. **Plans for disseminating and communicating study results, including the presence or absence of any restrictions on the extent and timing of publication**   We plan to submit our findings for publication in a peer-reviewed journal. No restrictions on the extent or timing of publication are present. This work forms part of an ESRC program grant (currently under review) which includes a work-package to design and curate tools and materials for the public, patients, clinicians and policy-makers to support initiatives on antimicrobial prescribing behaviour. |
| 1. **Limitations of the study design, data sources, and analytic methods**   The aim of this study is to make inferences about the effect of antibiotic treatment overall, and in different patient groups in order to develop models to support antibiotic prescribing decisions in primary care. The major challenge is to adjust for confounding by disease severity, i.e. the fact that patients with more severe infections are more likely to be prescribed an antibiotic. Estimating disease severity in primary care is challenging because clinical measures such as vital signs are not well recorded. We have outlined methods to try and account for this issue including the use of instrumental variables and treatment propensity scores.  This study will be limited to some extent in the outcomes it will be able to investigate. First, as shown in I. Sample size considerations, the power needed to identify differences in treatment effects by co-comorbidities will be limited by sample sizes available for extremely rare outcomes. However, as we have chosen our outcomes based on their importance for patients requesting antibiotics, we will look at more common outcomes like re-consultation for which sample sizes should be sufficient. The final effect sizes that we will be powered to detect will then depend on the actual number of observations for respiratory, urinary and skin/soft tissue infections. We will take this a priori power into account when discussing our findings. Secondly, we will rely on the representativeness of the data sources from which we obtain our outcome measures. In particular, HES A&E data coverage is incomplete in comparison to national A&E attendances. We expect this to bias our results for A&E consultations towards the null, limiting our power to detect true differences in outcomes. Again, this limitation will be taken into consideration when discussing any findings on A&E admissions.  By relying on clinical codes to define exposure and measure outcomes, this study may be subject to misclassification. It is known that considerable inter-practice variation exists in coding certain conditions. GPs may not use diagnostic codes to define every episode of e.g. URTI. We will aim to mitigate misclassification of URTIs and other infections by building on comprehensive code lists from previously published studies using CPRD and THIN and reviewing them with experts in the field (see Appendix Table 2). Even so, code lists may not always accurately represent the real reasons why antibiotics were prescribed, as much of the clinical information that is obtained during the consultation is not captured in them. However, one of the main aims of this study is to get a better understanding of this problem through linkage of prescribing and indication. This is further aided by exploring differences in coding between practices will be explored with novel methods, as described in *O. Plan for addressing confounding*.  Although the prescription data recorded in CPRD is of high quality, prescription records themselves do not guarantee that individuals will necessarily take the antibiotics. CPRD data describes antibiotics prescribed and it’s known that patients do not fill 100% of their prescriptions. With no valid way to estimate the proportion of prescribed CPRD antibiotics that were dispensed, we will be unable to ascertain which patients were adherent to the prescribed medications. Furthermore, the antibiotic prescription data used in this work is based on those prescribed from primary care, and thus does not take into account antibiotics prescribed from alternative healthcare sources, such as online, walk-in centres, and urgent care. |
| 1. **Amendments**   **Adding additional covariates in line with previous literature**  Summary:  We would like to include the following additional covariates:   - - Coronary Heart Disease   - Stroke   - Heart Failure   - Peripheral Arterial Disease   - Liver disease   - Immunosuppression   - Composite (Charlson Comorbidity Index - CCI)   Reasoning:  We originally excluded cardiovascular co-morbidities, liver disease, immunosuppression and the CCI because of insufficient power to detect our main effects of interest, i.e. differences between co-morbidities in the number needed to treat (*N. Data/ Statistical Analysis*, step 4.). While this is still the case, we now believe that we do need to include these variables as covariates in our analysis in order to ensure unbiased results and comparability to results from previous literature^1,16^. In line with the original protocol, **no** attempt will be made to interpret their effect sizes with regards to the number needed to treat (steps 4.-5.), as sample size calculations have indicated low power.  **Additional analysis of confounding**  Summary:  Preliminary analysis has shown unexpected differences to previously published results. These differences could be due to differences in datasets used, differences in countries analysed, or differences in practices and patients included. We plan to further investigate the reasons for the observed difference to be able to present the findings in context.  Reasoning:  After performing analysis of steps 1. and 2. described in section *N.* Data/ Statistical Analysis and comparing them to previous studies on THIN, we found differences in reported rates of antibiotic prescribing and rates of incidence of infection. The size of these differences was up to 30% and persists even when identical code lists and procedures are used. We are not entirely clear why we see these differences, but they seem unlikely to be chance findings and may be clinically relevant, thus warranting further investigation. All previous studies were performed using UK-wide, primary care-only data. We speculate that differences arose because we limited our sample to England-only, HES-linked data. For example, a recent study suggested that i) there might be differences in antibiotic prescribing behaviour between UK countries, with England having lower rates of prescribing than its neighbours.^16^ These conclusions are in line with our findings, but alternative explanations exist: ii) there might be differences between practices that agree to link their data to HES and ONS as compared to those who don’t, iii) there might be differences between patients who opt out of linkage as compared to those who don’t, and finally iv) there might be differences in practices that contribute to THIN compared to those that contribute to CPRD. In order to be able to appropriately interpret and use the results of this study, we aim to investigate which of the above hypotheses is the most likely. We will rerun all analysis in steps 1. and 2. (which use only primary care) on all patients in CPRD, using the definitions in *K. Study population* but omitting criteria concerning data linkage (bullet points a., c. and the second half of d.). The results from these calculations will then be used to judge whether the observed differences are due to practices excluded because of their country (i), due to practices excluded because of linkage (ii), due to patients excluded because of linkage (iii), or due to practices which are not in CPRD at all (iv). |
| 1. **References**   1. Shallcross L, Beckley N, Rait G, Hayward AC, Petersen I. Antibiotic prescribing frequency amongst patients in primary care: a cohort study using electronic health records. *J Antimicrob Chemother*. 2017;72(6):1818-1824.  2. English Surveillance Programme for Antimicrobial Utilization and Resistance. *Report 2015*.; 2015.  3. Public Health England. *Managing Common Infections: Guidance for Primary Care.*; 2014.  4. Hawker JI, Smith S, Smith GE, et al. Trends in antibiotic prescribing in primary care for clinical syndromes subject to national recommendations to reduce antibiotic resistance , UK 1995 – 2011 : analysis of a large database of primary care consultations. *J Antimicrob Chemother*. 2014;69(12).  5. Gulliford MC, Dregan A, Moore M V, et al. Continued high rates of antibiotic prescribing to adults with respiratory tract infection: survey of 568 UK general practices. *BMJ Open*. 2014;4(10).  6. Boggon R, Hubbard R, Smeeth L, et al. Variability of antibiotic prescribing in patients with chronic obstructive pulmonary disease exacerbations: a cohort study. *BMC Pulm Med*. 2013;13(1).  7. Rooshenas L, Wood F, Brookes-Howell L, Evans M. The influence of children’s day care on antibiotic seeking: a mixed methods study. *Br J Gen Pract*. 2014;64(622).  8. Little P, Hobbs FD, Moore M, et al. Clinical score and rapid antigen detection test to guide antibiotic use for sore throats: randomised controlled trial of PRISM (primary care streptococcal management). *BMJ*. 2013;347.  9. Francis NA, Butler CC, Hood K, Simpson S, Wood F, Nuttall J. Effect of using an interactive booklet about childhood respiratory tract infections in primary care consultations on reconsulting and antibiotic prescribing: a cluster randomised controlled trial. *BMJ*. 2009;339.  10. Public Health England. *Target Antibiotics Toolkit*.; 2014.  11. Petersen I, Johnson A, Islam A, Duckworth G, Livermore D, Hayward AC. Protective effect of antibiotics against serious complications of common respiratory tract infections: Retrospective cohort study with the UK General Practice Research Database. *BMJ*. 2007;335(7627).  12. Demidenko E. Sample size determination for logistic regression revisited. *Stat Med*. 2007;26(18).  13. Demidenko E. Sample size and optimal design for logistic regression with binary interaction. *Stat Med*. 2008;27(1).  14. Petersen I, Hayward AC, SACAR Surveillance Subgroup. Antibacterial prescribing in primary care. *J Antimicrob Chemother*. 2007;60 Suppl 1.  15. Booth HP, Prevost AT, Gulliford MC. Validity of smoking prevalence estimates from primary care electronic health records compared with national population survey data for England, 2007 to 2011. *Pharmacoepidemiol Drug Saf*. 2013;22(12).  16. Pouwels KB, Dolk FCK, Smith DRM, Smieszek T, Robotham J V. Explaining variation in antibiotic prescribing between general practices in the UK. *J Antimicrob Chemother*. 2018;73(Suppl. 2):ii27-ii35.  17. Schnabel RB, Sullivan LM, Levy D, et al. Development of a risk score for atrial fibrillation (Framingham Heart Study): a community-based cohort study. *Lancet*. 2009;373(9665).  18. Steyerberg EW, Vickers AJ, Cook NR, et al. Assessing the performance of prediction models: a framework for some traditional and novel measures. *Epidemiology*. 2010;21(1).  19. Austin PC, Stuart EA. Moving towards best practice when using inverse probability of treatment weighting (IPTW) using the propensity score to estimate causal treatment effects in observational studies. *Stat Med*. 2015;34(28).  20. Davies NM, Gunnell D, Thomas KH, Metcalfe C, Windmeijer F, Martin RM. Physicians’ prescribing preferences were a potential instrument for patients’ actual prescriptions of antidepressants(). *J Clin Epidemiol*. 2013;66(12).  21. McKay R, Mah A, Law MR, McGrail K, Patrick DM. Systematic Review of Factors Associated with Antibiotic Prescribing for Respiratory Tract Infections. *Antimicrob Agents Chemother*. 2016;60(7).  22. Sáez C, Robles M, García-Gómez JM. Stability metrics for multi-source biomedical data based on simplicial projections from probability distribution distances. *Stat Methods Med Res*. 2014;26(1).  23. Saez C, Zurriaga O, Perez-Panades J, Melchor I, Robles M, Garcia-Gomez JM. Applying probabilistic temporal and multisite data quality control methods to a public health mortality registry in Spain: a systematic approach to quality control of repositories. *J Am Med Inform Assoc*. 2016;23(6). |
| **List of Appendices** *(Submit all appendices as separate documents to this application)*   1. 170228_ISAC_appendix.xlsx 2. 170408_sample_size_calculations.docx |
