# Supplementary material for: Antibiotic prescribing for lower UTI in elderly patients in primary care and risk of bloodstream infection: A cohort study using electronic health records in England
Source: PLoS Med. 2020 Sep 21;17(9):e1003336. doi: 10.1371/journal.pmed.1003336 (PMC7505443; doi:10.1371/journal.pmed.1003336)
Supplement: S1 Text — BSI, bloodstream infection. (DOCX) [file pmed.1003336.s012.docx]

**S1 Text: Assertions of type of BSI.** BSI, bloodstream infection.

Bloodstream infection (BSI) in primary care was identified using Read codes. These codes usually represent the entry of hospital discharge information into the primary care record either by the General Practitioner or a nurse/administrator. BSI in secondary care was identified using International Classification of Diseases version 10 (ICD-10) codes for sepsis at hospital discharge based on an existing code list. For hospital-documented sepsis, the cause of sepsis was classified as urosepsis, sepsis of other infectious origin, and unspecified sepsis. Urosepsis was further divided into those that represented the main reason for admission (urinary tract infection (UTI) and sepsis code in positions 1&2) and secondary sepsis (codes in any other position). For sepsis due to other infections, defined as a sepsis code not linked to a UTI or urosepsis, we distinguished between respiratory and other causes of sepsis. Some sepsis cases were only reported in primary care. As it is highly unlikely that sepsis is managed entirely in primary care, we examined all ICD-10 codes relating to the patient’s hospital admission to try and understand the rationale for the primary care sepsis code.
