# Supplementary material for: Antibiotic prescribing for lower UTI in elderly patients in primary care and risk of bloodstream infection: A cohort study using electronic health records in England
Source: PLoS Med. 2020 Sep 21;17(9):e1003336. doi: 10.1371/journal.pmed.1003336 (PMC7505443; doi:10.1371/journal.pmed.1003336)
Supplement: Attachment — Submitted filename: Peer review UTI.docx [file pmed.1003336.s013.docx]

Peer review UTI

The authors offer a further examination of the issue of so called ‘delayed prescribing’ for adult UTI age >65 and its association with blood stream infection. This is an important topic to guide clinical practice. As the authors point out the diagnosis of UTI in the over 65s is more difficult and there are competing priorities regarding overuse of antibiotics (side effects and resistance) vs potential underuse (risk of complications including sepsis and blood stream infection). An earlier publication [1] suggested a 7-8 fold increase in the risk of BSI following delayed or no treatment. This publication however was controversial due to the definition of UTI episodes and imbalance between the patient characteristics. Plus the serious patient outcomes even in those treated with antibiotics suggests that something unusual was going on with patient selection. Furthermore I would suggest that delayed prescribing as described in the management of RTi is a positive management decision accompanied by information on natural history and safety net advice- usually accompanied by explicit means of access to a prescription. This is quite different to not prescribing but then prescribing later so the original authors[1] use of the term ‘delayed prescribing’ is in itself misleading. Prospective surveys in younger women with UTI show that 93-95%% will receive an antibiotic. [2, 3] Delayed prescribing has not been widely promoted as a management option for UTI although it has been tested in younger female adults in a clinical trial.[4]

In this paper the authors use the same data source (CPRD) but offer a more rigorous account of case definition. This is illustrated using a Figure (Figure 1) but I have to say I was none the wiser having examined the figure. I think I understand the case definition from the accompanying text but the figure itself was confusing to my mind. Patients with immediate adverse outcomes were also appropriately excluded since their disposition cannot have been influenced by the initial prescribing decision. The authors considered prescription in the following 7 days and non-prescription in the same group, this is appropriate for the reasons above (that this was unlikely a positive management option) and as they correctly point out delayed prescribing is not well coded in primary care records.

The issue with this data (in common with Ghabi) is that since delayed or non-treatment are not usual strategies for managing lower UTI in younger adults it seems unlikely that it is a usual strategy in a higher risk group (the over 65). Hence it is also probable that those not immediately treated are likely to be different from those offered immediately treatment. This was certainly the case with Ghabi and similarly there are baseline differences evident here. Those with immediate treatment are more likely to be female, are younger, are less deprived, more likely to have a prior history of both admission and antibiotic prescribing and more likely to be visited at home. Some of these differences are marked; female 80% vs 59%, antibiotic exposure 18% vs 27%, home visit 3.7% vs 9.6%. All of these factors were independently associated with risk of BSI so these differences are highly relevant to the analysis. Moreover overall this group has a poor outlook with 6% hospitalised within 60 days and 2% mortality- how does this compare with background rates in the population? In the adjusted analysis non use of antibiotics was not associated with BSI but was with excess mortality risk.

The authors appropriately summarise their findings and consider the limitations of the data which include issues with coding – other studies have demonstrated that many antibiotic prescriptions are not associated with appropriate coding – this analysis can only include those with a code who may differ from those un-coded in terms of symptom severity or certainty of diagnosis. There are also problems with coding of the outcome of interest and one third of sepsis codes were found only in the primary care record and may represent secular changes in coding styles.

The study findings were at odds with those reported by Gharbi but the question of safety of delayed prescribing remains uncertain. I think the authors could make clearer the difference between what is probably being investigated here (no or later prescribing) rather than an active policy to issue a deferred prescription as has been described and trialled for acute respiratory infection. It could be made more explicit that there may be fundamentally different management decisions being enacted due to different population characteristics. Age co-morbidity prior antibiotics and prior admission were all independent risk factors for BSI and are in themselves relevant findings for clinicians.

The authors call for a randomised trial to resolve the uncertainties but with an event rate of only 0.4% this would be a huge trial. Before proposing such a trial we need to better understand the prescribing decisions being made. Does this truly represent a positive strategy are other factors being taken into account. Is the excess mortality risk in those not receiving a prescription actually reflecting underlying risk and part of the decision making process? Is there an appetite in clinicians to offer delayed prescribing in this higher risk group- would they/patients agree to randomisation.

1.	Gharbi M, Drysdale JH, Lishman H, Goudie R, Molokhia M, Johnson AP, Holmes AH, Aylin P: **Antibiotic management of urinary tract infection in elderly patients in primary care and its association with bloodstream infections and all cause mortality: population based cohort study**. *BMJ* 2019, **364**:l525.

2.	Little P, Merriman R, Turner S, Rumsby K, Warner G, Lowes J, Smith H, Hawke C, Leydon G, Mullee M *et al*: **Presentation, pattern, and natural course of severe symptoms, and role of antibiotics and antibiotic resistance among patients presenting with suspected uncomplicated urinary tract infection in primary care: observational study**. *British Medical Journal* 2010, **340**.

3.	Butler CC, Francis N, Thomas-Jones E, Llor C, Bongard E, Moore M, Little P, Bates J, Lau M, Pickles T *et al*: **Variations in presentation, management, and patient outcomes of urinary tract infection: a prospective four-country primary care observational cohort study**. *Br J Gen Pract* 2017.

4.	Little P, Moore M, Turner S, Rumsby K, Warner G, Lowes J, Smith H, Hawke C, Leydon G, Arscott A *et al*: **Effectiveness of five different approaches in management of urinary tract infection: randomised controlled trial**. *British Medical Journal* 2010, **340**.
