# Supplementary material for: Antibiotic prescribing for lower UTI in elderly patients in primary care and risk of bloodstream infection: A cohort study using electronic health records in England
Source: PLoS Med. 2020 Sep 21;17(9):e1003336. doi: 10.1371/journal.pmed.1003336 (PMC7505443; doi:10.1371/journal.pmed.1003336)
Supplement: Attachment — Submitted filename: UTI_BSI_author_reply.docx [file pmed.1003336.s014.docx]

**RE: PMEDICINE-D-20-00884R1: manuscript "Antibiotic prescribing for lower UTI in elderly patients in primary care and risk of bloodstream infection: a cohort study using electronic health records"**

## Comments from reviewer #1

**Comment #1.1:** Year of the episode is very important for capturing the time trend/effect on the association. In table 2, it's a bit odd to set the middle year of 2010/11 as benchmark. To be neat and conventional, it would be better to group them by every two years into 4 cohorts and use the first cohort (2007 and 2008) as benchmark for comparison. This will apply for all the analyses in the supplementary tables. Also, to be consistent, the breakdown of year of episode needs to appear in table 1 too.

**Author response:**

We changed the reference year to 2007/08 in all analyses and added a summary of year to Table 1. We decided against grouping years into intervals of 2 years as suggested by the reviewer in order to allow for a direct comparison with Gharbi et al. (2019). We believe this is justified by the large number of patients in the first year (>30,000), which provide a sufficient baseline group. A sensitivity analysis of grouping years into intervals of 2 years also did not show any notable difference in estimated effect sizes.

**Comment #1.2:** In statistical methods on page 10, it says "continuous variables were summarised using means and standard deviations, and categorical variables using absolute numbers and proportions. Wilcoxon rank tests (continuous) and tests (categorical) were used to assess the difference between exposure groups". However, for continuous variables in table 1, it depends on the distribution of the data, for those with normal distribution such as age, they should be summarised using mean and SD and compared with t-test. For those with non-normal (skewed) distribution such as CCI, Number of weeks spent in hospital in prior year, Number of admissions in prior year, and Number of attendances in prior year, they should be summarised using median and IQR and compared using Wilcoxon rank-sum test. In table 1 in the row for Number of attendances in prior year (15,142 (5·4) 0·44 (1·0) 0·53 (1·2) <0·001), the value of 15,142 (5.4) is very strange. Is it a typo?

**Author response:**

We agree with the reviewer that normally distributed variables should usually be summarised using mean/standard deviation and compared with t-tests, whereas skewed variables should be summarised using median/interquartile range (IQR) and compared using a rank test.

However, we believe that our use of means and standard deviations is justified in our analysis because most of our continuous variables were count data (number of hospital visits, number of A&E visits etc.), and the majority of our patients have a record of 0. For example, more than 75% of patients did *not* have a hospital visit in the year prior to episode start. As a result, the median/IQR is uninformative for most continuous variables since it is equal to 0 [0-0] for all count variables except Charlson Comorbidity index. We have therefore continued to summarise continuous variables using mean and standard deviation but have added a footnote.

If the reviewer or editors have a strong opinion that the variables should nevertheless be summarised using median/IQR, we are happy to change the information in Table 1 accordingly.

Similarly, after careful deliberation we decided to continue using the Wilcoxon rank-sum test for all continuous variables. While we agree with the reviewer that a t-tests would be preferable for normally distributed data, none of our continuous variables exhibited an approximate normal distribution. Due to the demographic population structure and the inclusion criteria applied to our cohort, even age followed a decidedly non-normal distribution in our analysis (it was roughly uniformly distributed up to age 80 and then exhibited a skew up to age 105; see figure below). We therefore believe that the choice to use a Wilcoxon rank-sum test provides the most appropriate and conservative estimates. The main advantage of using a t-test would be increased statistical power to detect true effects if its assumptions are met. Since differences in age, Charlson Comorbidity Index and prior hospital activity were all found to be highly statistically significant even under the non-parametric test, no substantial difference in conclusions would be expected from a parametric t-test. We did add the suffix “-sum” to the text to distinguish the Wilcoxon rank-sum test used in our study from the similarly named but inappropriate Wilcoxon signed-rank test.

The number 15,142 (5·4) was indeed a typo and we thank the reviewer for the thorough review of the table. The correct number was 0.45 (1.1).

**Comment #1.3:** As pointed out above that a few variables are non-normally distributed, they need to be transformed (maybe using log transformation) into normal variables in the GEE analyses in table 2. This also applies to the other regression analyses in the supplementary tables.

**Author response:**

Since all of our skewed variables except age were counts that included 0, we decided to use a square root transformation. This decision was also supported by a comparison of the information criterions, which favoured the square root over both log(x + 1) and log(x + 0.1). In the revised analysis, all count variables were transformed in this way. Age was not transformed since the transformation was judged inappropriate both by visual inspection and by information criteria. We added a sentence describing this transformation to the statistical analysis section:

*All count variables (CCI, number of admissions, number of days spent in hospital, and number of attendances) were transformed using the square root before adding them to the GEEs.*

The substantial skew of most of our continuous variables meant that even after transforming them using the square root (or any other power transformation covered by a Box-Cox transformation) none exhibited a normal distribution. We would like to note at this point that normality of predictors is not an assumption of logistic regression or GEEs. Instead, we aim for linearity in the relationship with the outcome, which appears to be reasonably satisfied by a square-root transformation (see example figures below for the linear relationship between risk of sepsis and grouped continuous predictors).

**Age:**

**Charlson comorbidity index** (square root):

**Number of hospital admissions in prior year:**

**Comment #1.4:** As delayed antibiotic prescribing is not well recorded in electronic health records, the authors considered patients who were not prescribed antibiotics and those with a delayed prescription as a single group. This becomes inaccurate and may lead to biased results. The authors need to discuss the impact of this in the limitation and also need to tone down the claims on delayed antibiotic on mortality as the data is imprecise.

**Author response:**

We added some further comments to the explanation around coding of delayed prescribing in the discussion as follows:

“Since delayed prescribing is not well recorded in primary care, it is feasible that some individuals recorded as receiving antibiotics immediately actually received a delayed prescription. *Depending on the extent of misclassification between treatment groups, this may have led us to under-estimate the reported association between antibiotic treatment and adverse outcomes. However, whereas delayed prescribing is commonly used for respiratory tract infections, evidence to support this approach for UTI is limited. For these reasons, it seems likely that most who were prescribed antibiotics on the date of their consultation started treatment on the same date. Conversely, if there were substantial differences in outcome between delayed prescribing and no prescribing, treating them as one group may over-estimate the reported association between delayed prescribing and mortality, although the results reported by Gharbi et al.* [*[13]*](https://paperpile.com/c/aMDzPB/Mb2O) *suggests that this is not the case.”*

We have also toned down our interpretation of the association between mortality and delayed prescribing as follows:

Paragraph 2 of the discussion changed to:

*Although we found some evidence that individuals who were not prescribed antibiotics immediately were at increased risk of death, these results need to be interpreted with some caution since they may be subject to bias and residual confounding.*

We also added the following to the discussion:

“*Recognising the limitations of analyses based on routine data*, we find no evidence of an association between delaying or withholding antibiotics and bloodstream infection, but some evidence of increased mortality.

## Comments from reviewer #2

**Comment #2.1:** there are two relatively recent primary care trials in uncomplicated urinary tract infections (Gagyor et al, your reference 24 and Vik I et al, PLOSMedicine 2018), both showing a clear beneficial effect in women and also less complications (fever, pyelonefritis). So in which elderly patients do we need a placebo controlled trial as you suggest in your discussion? Not in the elderly with a true urinary tract infection I should think? And in those with asymptomatic bacteriuria we do not want a trial either? So, the real challenge is to detect true urinary infection? The authors should discuss this in my opinion.

**Author response:**

Thanks to the reviewer for raising this. In light of these comments and those of reviewer 3 we have removed the recommendation for a trial and instead emphasised the need for novel diagnostic / risk prediction strategies to guide antibiotic prescribing decisions for suspected UTI, and also to quantify benefits and harms. The concluding paragraph has been amended as follows:

“The safety of delaying or withholding antibiotics in adults aged ≥65 years with suspected UTI is uncertain. *Adverse consequences of antibiotic treatment in this population and the public health imperative to tackle antibiotic resistance highlight the need for novel diagnostic and/or risk prediction strategies to guide antibiotic prescribing decisions for suspected UTI.*

**Comment #2.2:** Line 84: the percentage of overprescription depends heavily on the quality of diagnosis and varies across settings and countries, see Butler C et al, Brit J Gen Pract 2017

**Author response:**

We have added the following to this sentence to highlight this point:

“An estimated 40-50% of antibiotic prescriptions for UTI are estimated to be inappropriate,[[9]](https://paperpile.com/c/YApu0u/iM7A1) *although the degree of inappropriate prescribing varies widely across settings and countries (Butler et al. 2017).”*

**Comment #2.3:** Line 85: reduction in prescribing was mainly achieved in respiratory infections

**Author response:**

We have added the following to the introduction:

“A wide range of national initiatives aiming to tackle inappropriate prescribing have reduced total prescribing by 13.2% between 2013 and 2017,[[10]](https://paperpile.com/c/YApu0u/kheZQ) *mainly by reducing prescribing for respiratory tract infections. This has reduced total prescribing of broad-spectrum antibiotics, even in elderly populations.[11]*

**Comment #2.4:** Line 204: some comorbidities are much more important than others in terms of risk for complicated infections (for instance cardiac failure versus hip arthritis). Was this taken into account?

**Author response:**

We agree with the reviewer that risk of infectious complications varies by comorbidity. However, as comorbidities were not our main risk factor of interest we believe that adjusting for the Charlson Comorbidity Index is a simple method to adjust for confounding by comorbidity, with the advantage is that it allows us to directly compare our results with those of Gharbi et al. At the time of data collection Public Health England recommended more cautious antibiotic prescribing in primary care for asthma, chronic kidney disease (CKD), chronic obstructive pulmonary disease (COPD), diabetes, heart failure, immunosuppression, liver disease, peripheral arterial disease (PAD), and stroke. With exception of asthma, all of these are covered by the Charlson Comorbidity Index and they make up a majority of the index score.

It is extremely challenging to estimate the impact that different comorbidities may have on susceptibility to infection, particularly when using routine data. We have added a sentence to the discussion to acknowledge the limitations of our approach, as follows:

*Finally, we used the Charlson Comorbidity index as a composite measure of comorbidity. This had the advantage of making our analysis comparable with* [*Gharbi et al. 2019)*](https://paperpile.com/c/aMDzPB/Mb2O)*., but it does not take account of the fact that specific comorbidities, such as those affecting the urogenital tract, may impact an individuals’ risk of bacteraemia more than others (and therefore influence GPs’ prescribing decisions.)*

**Comment #2.5:** An important residual confounder could be that patients did not want to be treated because they had accepted end of life? These elderly patients do not always have a clear higher frailty score than those who full of good spirit?

**Author response:**

A sentence discussing this confounder and the risk of residual confounding more generally was added to the discussion as follows:

*Remaining differences in patient characteristics between groups suggest that we were unable to fully account for confounders. We conclude that residual confounding persists, likely due to factors that influence GPs prescribing decisions but are not well recorded in EHR data, such as severity of clinical presentation, knowledge of patient preferences, for example in relation to end of life care, and the patient’s social circumstances.*

## Comments from reviewer #3

**Comment #3.1:** In this paper the authors use the same data source (CPRD) but offer a more rigorous account of case definition. This is illustrated using a Figure (Figure 1) but I have to say I was none the wiser having examined the figure. I think I understand the case definition from the accompanying text but the figure itself was confusing to my mind.

**Author response:**

We simplified Figure 1 and its legend to improve its readability.

**Comment #3.2:** The issue with this data (in common with Ghabi) is that since delayed or non-treatment are not usual strategies for managing lower UTI in younger adults it seems unlikely that it is a usual strategy in a higher risk group (the over 65). Hence it is also probable that those not immediately treated are likely to be different from those offered immediately treatment. This was certainly the case with Ghabi and similarly there are baseline differences evident here. Those with immediate treatment are more likely to be female, are younger, are less deprived, more likely to have a prior history of both admission and antibiotic prescribing and more likely to be visited at home. Some of these differences are marked; female 80% vs 59%, antibiotic exposure 18% vs 27%, home visit 3.7% vs 9.6%. All of these factors were independently associated with risk of BSI so these differences are highly relevant to the analysis. Moreover overall this group has a poor outlook with 6% hospitalised within 60 days and 2% mortality- how does this compare with background rates in the population?

**Author response:**

According to official statistics by the UK Office for National Statistics (ONS), the background yearly mortality rate for patients aged 65-105 ranges from 1-22%, resulting in an average yearly mortality of 4.3%. This translates to a bimonthly rate of just under 1%. Since our study has a higher proportion of patients aged >75 and all of our patients are actively consulting for an infectious condition, we believe our slightly higher reported mortality rate of 2% is plausible.

We do not have similar numbers for hospitalisation rates by age, but given the magnitude of the mortality rate we believe that a hospitalisation rate within 60 days of 6% is also within the range of reason.

References to mortality and population count statistics:

<https://www.ons.gov.uk/file?uri=%2fpeoplepopulationandcommunity%2fbirthsdeathsandmarriages%2fdeaths%2fdatasets%2fdeathregistrationssummarytablesenglandandwalesdeathsbysingleyearofagetables%2f2016/deathsyoauk2016tablesfinal.xls>

<https://www.ons.gov.uk/file?uri=/peoplepopulationandcommunity/populationandmigration/populationestimates/datasets/populationestimatesforukenglandandwalesscotlandandnorthernireland/mid2015/ukandregionalpopulationestimates18382015.zip>

We completely agree with the reviewers overall point that there appears to be a systematic difference between individuals who are prescribed antibiotics immediately and those that are not. We have commented on this in more detail in the discussion - please see response to point 3.3 below

**Comment #3.3:** The study findings were at odds with those reported by Gharbi but the question of safety of delayed prescribing remains uncertain. I think the authors could make clearer the difference between what is probably being investigated here (no or later prescribing) rather than an active policy to issue a deferred prescription as has been described and trialled for acute respiratory infection. It could be made more explicit that there may be fundamentally different management decisions being enacted due to different population characteristics. Age co-morbidity prior antibiotics and prior admission were all independent risk factors for BSI and are in themselves relevant findings for clinicians.

**Author response:**

We have added the following to the discussion paragraph 2 to try and explain the issues highlighted in the paragraph above:

Individuals who were not treated with antibiotics immediately were more likely to be admitted to hospital for reasons unrelated to UTI or BSI, which implies that the risk of BSI and death in some patients included in this study may have been driven by a delay in diagnosing the patient’s underlying illness, rather than a delay in initiating antibiotics for lower UTI. *This hypothesis is supported by the finding of systematic differences between patients who were or were not treated immediately with antibiotics across a range of factors (comorbidity, antibiotic use in the prior 30 days) that might influence risk of BSI. Taken together, this provides some evidence that antibiotic prescribing decisions may have been influenced by factors (which are not evident in EHRs) that are unrelated to antibiotic treatment decisions for suspected UTI.*

We have also changed the first sentence of the discussion to make it clearer that this is not an evaluation of delayed prescribing:

“In this study, *individuals who were not treated immediately with antibiotics (on the date of their initial GP consultation) for suspected UTI* were not at increased risk of BSI.”

**Comment #3.4:** The authors call for a randomised trial to resolve the uncertainties but with an event rate of only 0.4% this would be a huge trial. Before proposing such a trial we need to better understand the prescribing decisions being made. Does this truly represent a positive strategy are other factors being taken into account. Is the excess mortality risk in those not receiving a prescription actually reflecting underlying risk and part of the decision making process? Is there an appetite in clinicians to offer delayed prescribing in this higher risk group- would they/patients agree to randomisation.

**Author response:**

Thank you for your helpful feedback. We take the point that it would require an extremely large trial and this should be preceded by work to understand whether a strategy of delayed prescribing for UTI would be acceptable to patients and clinicians. We have therefore removed the recommendation for a trial from the abstract and conclusions and made the following changes:

a) This sentence to the discussion:

*“However, it is unclear whether use of delayed prescribing for suspected UTI would be acceptable to GPs or to patients in this age-group.”*

b) We have re-worded the section on clinical /policy implications:

This population-based study *highlights uncertainty around the risks and benefits of antibiotic treatment for suspected UTI in patients aged ≥65 years. The increased risk of adverse outcomes in this age-group may make GPs more likely to prescribe antibiotics.*

c) We have changed the conclusion as follows:

The safety of delaying or withholding antibiotics in adults aged ≥65 years with suspected UTI is uncertain. *Adverse consequences of antibiotic treatment in this population and the public health need to tackle antibiotic resistance highlight the need for novel diagnostic and/or risk prediction strategies to guide antibiotic prescribing decisions for suspected UTI.*

## Requests by the editors

**Request #1:** For some reason, the paper title entered as metadata into our submission system mentions antibiotic prescription for lower respiratory tract infection rather than UTI as the exposure variable, which is a bit confusing - if you are able to correct it when submitting the revision, that would be great.

**Author response:**

This was an unintentional mistake due to concurrent studies by the authors dealing with respiratory tract infections. This has now been corrected.

**Request #2:** At this stage, we ask that you include a short, non-technical Author Summary of your research to make findings accessible to a wide audience that includes both scientists and non-scientists. The Author Summary should immediately follow the Abstract in your revised manuscript. This text is subject to editorial change and should be distinct from the scientific abstract.

**Author response:**

An Author Summary accessible for a lay audience has been added following the guidance provided in the author guidelines.

**Request #3:** In the last sentence of the Abstract Methods and Findings section, please add a note summarising any key limitation(s) of the study's methodology.

**Author response:**

A note on key limitations has been added to the Methods and Findings section in the abstract:

*“Limitations include overlap between the categories of immediate and delayed antibiotic prescribing, residual confounding underlying differences between patients who were/were not treated with antibiotics, and lack of microbiological diagnosis for BSI.”*

**Request #4:** We'd recommend stating in the Methods paper whether the analytical approach described in this paper corresponded to that laid out in a prospective protocol or analysis plan? Please state this (either way) early in the Methods section.

**Author response:**

A reference to the analysis protocol submitted for ethical approval is included in the first subsection of the Methods section:

*This study was conducted as part of the Preserving Antibiotics through Safe Stewardship (PASS) project.*[*[17]*](https://paperpile.com/c/aMDzPB/3Fn9) *Access to CPRD data within PASS* was approved by the Medicines and Healthcare products Regulatory Agency (MHRA UK) Independent Scientific Advisory Committee [ISAC-Nr.: 17 048], under Section 251 (NHS Social Care Act 2006).

We added another statement to the statistical analysis section stating that the definitions were adjusted to mirror those employed by Gharbi et al. as closely as possible to allow for comparison.

*The analysis presented here was outlined prospectively in the protocol submitted to the* MHRA *Independent Scientific Advisory Committee for ethical approval (see supplementary material). The definitions and methods were chosen to replicate the analysis performed by Gharbi et al.* [*[13]*](https://paperpile.com/c/aMDzPB/Mb2O) *as closely as possible and to address all concerns raised by researchers regarding the validity of those findings.* [*[14]*](https://paperpile.com/c/aMDzPB/FcUI)

Finally, we uploaded the analysis protocol together with the revised manuscript.

**Request #5:** The authors might wish to consider using a formal reporting guideline to enhance reporting of their study, before submitting the revision. Choices for this work might include STROBE (for epidemiological studies) or RECORD (for studies involving routinely-collected data).

**Author response:**

A filled-in RECORD reporting guideline has been added to the submission.
