# Supplementary material for: Antibiotic prescribing for lower UTI in elderly patients in primary care and risk of bloodstream infection: A cohort study using electronic health records in England
Source: PLoS Med. 2020 Sep 21;17(9):e1003336. doi: 10.1371/journal.pmed.1003336 (PMC7505443; doi:10.1371/journal.pmed.1003336)
Supplement: Attachment — Submitted filename: UTI_BSI_author_reply2.docx [file pmed.1003336.s015.docx]

July 30^th^, 2020

**RE: PMEDICINE-D-20-00884R2: manuscript "** **Antibiotic prescribing for lower UTI in elderly patients in primary care and risk of bloodstream infection: a cohort study using electronic health records"**

Dear Dr Moyer,

We are grateful that you found our manuscript acceptable for publication in PLOS Medicine. On behalf of our co-authors, we have submitted a revised manuscript via the online portal. All of the authors named on the paper have reviewed and approved this revised manuscript.

In the pages following this letter, we describe all changes made in response to requests by the editorial board and the reviewers. We also ensured that our manuscript now conforms with PLOS Medicine’s submission guidelines, addressing all issues highlighted by the production team.

Yours sincerely,

Laura Shallcross & Patrick Rockenschaub

Laura Shallcross, PhD (Corresponding Author)

Associate Professor

Institute of Health Informatics

University College London

222 Euston Rd, NW1 2AB, London, UK

## Editorial requests

Please find our response to some of the requested changes below. Where requests are not explicitly listed, changes were unambiguous and made exactly as suggested.

**Request #12:** Results, page 16: Days to BSI diagnosis findings- Please reference the table where these are presented, and please provide p values in the text.

**Author response:**

Days to BSI diagnosis originally were a standalone calculation and were not presented in any of the included tables. We added them to Table 1, where we think the results fit best and added a p-value to the text.

**Request #16:** Discussion: Please present and organize the Discussion as follows: a short, clear summary of the article's findings; what the study adds to existing research and where and why the results may differ from previous research; strengths and limitations of the study; implications and next steps for research, clinical practice, and/or public policy; one-paragraph conclusion.

**Author response:**

We switched the order of the sections “Comparison with existing literature” and “Strengths and limitations of this study” to comply with the suggested Discussion structure.

**Request #18:** RECORD checklist: Thank you for including the RECORD checklist.  Some of the items have been left blank- please ensure that the checklist is completed.

**Author response:**

We thoroughly read over the RECORD checklist and were unfortunately not able to find items that had been left blank. Could the editors please indicate which items were left blank?

**Request #21:** Table 1: In the legend, please define abbreviations for UTI, BSI, CCI, OR, and SD.

**Author response:**

We added all requested abbreviations to the table legend with the exception of OR, which was not presented in this table. Following the change requested by reviewer 1 (see below), an abbreviation for IQR instead of SD was also included. We additionally defined the abbreviations IMD, Q1-Q5, and A&E to ensure consistency in our use of abbreviations. The same was done for Table 2 (Request #22).

**Requests #24-26:** Supporting information Table 2-4: Please provide the unadjusted OR (with 95% CIs and p values) in addition to the adjusted OR.  Please note in the legend the variables that were adjusted for in the adjusted analysis. Please define abbreviations for OR, CI, BSI, UTI, NHS in the figure legend.

**Author response:**

We added unadjusted ORs to each of these tables. Unadjusted ORs were originally omitted due to space restrictions. To ensure the readability of each table, Supporting information Tables 2-4 were split up into 6 separate tables (new Supporting information Tables 2-7). Original Supporting information Tables 5-6 became new Supporting information Tables 8-9.

## Comments from reviewer #1

**Comment:** Thanks authors for their effort to improve the manuscript. However, I am still not satisfied with the response for comment 1.2 on the summary of data in table 1. The authors' argument doesn't make sense at all. It is very simple rule that normal data should be summarised as mean and SD and non-normal data as median and IQR, and followed by proper t-test or non-parametric test like Wilcoxon rank-sum test. I insist that authors must follow this rule like all the other PLOS Medicine authors to correct this in the paper. By the way, all the other comments were well addressed so it's fine.

**Author response:**

We apologies if our original response was long-winded and not easily understandable. If we understand the reviewer correctly, then he/she requests that we use mean/SD for normally distributed variables and median/IQR for non-normally distributed variables. Since none of our continuous variables exhibits approximate normality (see response to previous comment #1.2), we conclude that the reviewer requests that we should change all summary and comparisons of continuous variables to median/IQR and Wilcoxon rank-sum tests.

We made the changes to Table 1 as requested by the reviewer.

## Comments from reviewer #3

**Comment:** I think you could add one further bullet to the section what did the reviewers do and find the positive statement reflecting the independent risk factors associated with BSI (below)
Women were less likely to develop BSI compared to men (OR 0.49, 95% CI: 0.43-0.55).
Increasing age (OR 1.22, 95% CI: 1.18-1.27 per 5 years) and social deprivation (Q5 versus
Q1: 1.45; 95%-CI: 1.19-1.76) were independently associated with BSI.

**Author response:**

The bullet point was added as suggested by the reviewer.
